# Safe management of bodies of deceased persons with suspected or confirmed COVID-19: a rapid systematic review

**DOI:** 10.1136/bmjgh-2020-002650

**Published:** 2020-05-14

**Authors:** Sally Yaacoub, Holger J Schünemann, Joanne Khabsa, Amena El-Harakeh, Assem M Khamis, Fatimah Chamseddine, Rayane El Khoury, Zahra Saad, Layal Hneiny, Carlos Cuello Garcia, Giovanna Elsa Ute Muti-Schünemann, Antonio Bognanni, Chen Chen, Guang Chen, Yuan Zhang, Hong Zhao, Pierre Abi Hanna, Mark Loeb, Thomas Piggott, Marge Reinap, Nesrine Rizk, Rosa Stalteri, Stephanie Duda, Karla Solo, Derek K Chu, Elie A Akl, the COVID-19 Systematic Urgent Reviews Group Effort (SURGE) group

**Affiliations:** 1 Clinical Research Institute, American University of Beirut, Beirut, Lebanon; 2 WHO Collaborating Centre for Infectious Diseases, McMaster University, Hamilton, Ontario, Canada; 3 Research Methods and Recommendations and the Michael G. DeGroote Cochrane Canada and McGRADE Centres, McMaster University, Hamilton, Ontario, Canada; 4 Hull York Medical School, University of Hull, Hull, UK; 5 Global Evidence Synthesis Initiative, American University of Beirut, Beirut, Lebanon; 6 University Libraries, Saab Medical Library, American University of Beirut, Beirut, Lebanon; 7 Department of Health Research Methods, Evidence and Impact, McMaster University, Hamilton, Ontario, Canada; 8 Medical School, Vita Salute San Raffaele University, Milano, Italy; 9 Guangzhou University of Chinese Medicine, The Fourth Clinical Medical College, Guangdong, China; 10 Beijing University of Chinese Medicine, Dongzhimen Hospital, Beijing, China; 11 Institute of Acupuncture and Moxibustion, China Academy of Chinese Medical Sciences, Beijing, China; 12 Infectious Disease Division, Rafik Hariri University Hospital, Beirut, Lebanon; 13 Department of Pathology and Molecular Medicine, McMaster University, Hamilton, Ontario, Canada; 14 London School of Hygiene and Tropical Medicine, London, UK; 15 Division of Infectious Diseases, American University of Beirut, Beirut, Lebanon; 16 Department of Internal Medicine, American University of Beirut, Beirut, Lebanon

**Keywords:** systematic review, public health, prevention strategies, respiratory infections

## Abstract

**Introduction:**

Proper strategies to minimise the risk of infection in individuals handling the bodies of deceased persons infected with 2019 novel coronavirus (2019-nCoV) are urgently needed. The objective of this study was to systematically review the literature to scope and assess the effects of specific strategies for the management of the bodies.

**Methods:**

We searched five general, three Chinese and four coronavirus disease (COVID-19)–specific electronic databases. We searched registries of clinical trials, websites of governmental and other relevant organisations, reference lists of the included papers and relevant systematic reviews, and Epistemonikos for relevant systematic reviews. We included guidance documents providing practical advice on the handling of bodies of deceased persons with suspected or confirmed COVID-19. Then, we sought primary evidence of any study design reporting on the efficacy and safety of the identified strategies in coronaviruses. We included evidence relevant to contextual factors (ie, acceptability). A single reviewer extracted data using a pilot-tested form and graded the certainty of the evidence using the GRADE approach. A second reviewer verified the data and assessments.

**Results:**

We identified one study proposing an uncommon strategy for autopsies for patients with severe acute respiratory syndrome. The study provided very low-certainty evidence that it reduced the risk of transmission. We identified 23 guidance documents providing practical advice on the steps of handling the bodies: preparation, packing, and others and advice related to both the handling of the dead bodies and the use of personal protective equipment by individuals handling them. We did not identify COVID-19 evidence relevant to any of these steps.

**Conclusion:**

While a substantive number of guidance documents propose specific strategies, we identified no study providing direct evidence for the effects of any of those strategies. While this review highlights major research gaps, it allows interested entities to build their own guidance.

**Summary box**

**What is already known?**

- There is scarce evidence on the transmission of coronavirus disease 2019 (COVID-19) and other coronaviruses from the dead bodies of confirmed or suspected cases.
- There are uncertainties about how to handle the bodies of deceased persons with confirmed or suspected COVID-19.

**What are the new findings?**

- The main findings of this review are based on guidance documents as opposed to primary studies.
- We identified one eligible study that evaluated a biosafety level 3 laboratory for autopsies of patients with SARS.
- We summarised the steps from 23 guidance documents providing practical advice on the steps of handling the bodies and the use of personal protective equipment by individuals handling them.

**What do the new findings imply?**

- WHO will use the findings of this study to inform its response to the COVID-19 pandemic.
- The interpretation of the findings needs to consider that the included guidance are not supported by direct evidence.
- There are additional research questions on the modes of transmission of COVID-19 from dead bodies to individuals, the desirable and undesirable effects of each management strategy, and contextual factors that require evidence.

## Introduction

On 11 March, WHO characterised the 2019 novel coronavirus (2019-nCoV) outbreak as a pandemic. On 20 April 2020, the pandemic had affected more than 2 400 000 individuals worldwide. Over the same time period, it has claimed the lives of 165 000 individuals.^1^ More concerning than the absolute number of deaths is their rate of increase.

The severe acute respiratory syndrome coronavirus 2 (SARS-CoV-2), which causes the coronavirus disease 2019 (COVID-19), has been characterised as highly contagious, with a relatively high case fatality rate, compared with other viral respiratory infections. This has created anxiety about the preparation and the burial of the bodies of deceased persons with suspected or confirmed COVID-19. There appears to be scarce evidence on the transmission of COVID-19 and other coronaviruses from the dead bodies of confirmed or suspected cases. In one study, Liu *et al* reported that 27.8% of healthcare workers in contact with deceased cases of severe acute respiratory syndrome (SARS) contracted the coronavirus.^2^ In another study, Mahallawi reported a case of Middle East respiratory syndrome (MERS) coronavirus in nasal secretions of a dead case.^3^ Similarly, there is confusion about how to safely perform autopsies on those bodies. Considering these uncertainties about how to handle the bodies of deceased persons with confirmed or suspected COVID-19 cases, answers to these questions are on the priority list for WHO.

Individuals at risk include healthcare workers, morgue staff, transport staff, family members, crematories staff, burial staff and religious staff. It is also a cultural challenge, as some cultural aspects and religious practices may influence how the bodies are handled and the associated risk of transmission. Moreover, the acceptability of different management strategies might vary across cultural and religious groups.

The objective of this study was to systematically review the literature to first scope, and then assess the effects of, specific strategies for the management of the bodies of deceased persons with suspected or confirmed COVID-19. We conducted a rapid systematic review, commissioned by WHO, to be able to inform their response to COVID-19 pandemic.

## Methods

The protocol was submitted to PROSPERO; however, it was not accepted because of the scoping component. We have registered the protocol in Open Science Framework (https://osf.io/j3nft).

### Search strategy and selection criteria

We conducted a rapid systematic review to identify, select, abstract, assess, and synthesise the available evidence addressing our question of interest.^4^

We developed the search strategy with the assistance of an information specialist experienced with systematic reviews (LH). Two information specialists peer reviewed the search strategy. Other members of the team, particularly the content experts, provided feedback to the search strategy. An additional search strategy was developed to identify indirect evidence from systematic reviews on SARS and MERS. We searched the following general electronic databases: Medline (using OVID platform), PubMed, EMBASE, CINAHL (using OVID platform) and the Cochrane Library. We also searched COVID-19-specific electronic databases such as COVID-19 Open Research Dataset (CORD-19), COVID-19 Research Database maintained by WHO (including its daily updates), Epistemonikos COVID-19 L·OVE platform and EPPI Centre living systematic map of the evidence. In addition, we searched Chinese databases such as WHO Chinese database, CNKI and China Biomedical Literature Service. Online supplementary appendix 1 includes the search strategies for the different general databases. The search strategies combined medical subject headings (MeSH) and keywords for the two following concepts: COVID-19 and dead bodies. PubMed search terms were informed by the Biomedical Information of the Dutch Library Association specialists curated search blocks.^5^ The related searches covered the date range from inception to 26 March 2020. We used no language restrictions.

10.1136/bmjgh-2020-002650.supp1 Supplementary data

Additional searches included searching for registered clinical trials in both the U.S. National Library of Medicine Register of Clinical Trials (ClinicalTrials.gov) and the WHO International Clinical Trials Registry Platform, searching for relevant documents on the websites of governmental and other relevant organisations, screening reference lists of the included papers and relevant systematic reviews, and searching Epistemonikos for relevant systematic reviews addressing SARS and MERS.

We included studies meeting specific criteria for the population, interventions, comparisons and outcomes of interest. Our populations of interest included the bodies of deceased persons with suspected or confirmed COVID-19, and the individuals handling those bodies, including nursing and medical personnel, morgue staff, transport staff, family members, crematories staff, burial staff and religious staff. In addition, and as a source of indirect evidence for primary studies, we considered primary studies and systematic reviews about the bodies of deceased persons with suspected or confirmed infections with either SARS virus or the MERS virus, as well as the individuals handling those bodies. The interventions of interest included any strategy to manage bodies of deceased persons, during the different phases of the process. These strategies would address either the bodies themselves or the individuals handling them. The outcomes of interest included risk of COVID-19 transmission to the individuals handling the bodies and to members of the community, morbidity and mortality associated with COVID-19, unintended harms of the management strategies, acceptability by different stakeholders (family members of the deceased person, members of the community, individuals handling the dead bodies, health authorities), and surrogate outcomes such as contact or droplet transmission. Also eligible were studies providing evidence relevant to contextual factors such as acceptability, feasibility, impact on equity and resources considerations related to the interventions of interest.

We included any study design including randomised controlled trials, non-randomised studies (including cohort studies, case–control studies, case series and case reports) and qualitative studies. In addition, we included guidance documents on the handling of bodies of deceased persons with suspected or confirmed COVID-19 identified from the websites of relevant organisations and national authorities by content experts and peer-reviewed literature.

### Study selection

We exported the literature search results to EndNote X9 for de-duplication, then to Covidence software. All reviewers pilot-tested a standardised title and abstract screening form using the same 30 citations. Once the form was calibrated, the reviewers screened in duplicate and independently all titles and abstracts using above listed eligibility criteria. We obtained the full texts for citations judged as potentially eligible by either reviewer.

All reviewers pilot-tested a full-text screening form using the same five full-text articles. Once the form was calibrated, the reviewers screened the full texts independently and in duplicate and resolved any conflicts by discussion, or with the help of a third reviewer. We recorded the primary reason for exclusion at the full-text screening stage.

### Data extraction

We developed and piloted with all reviewers a standardised data abstraction form in Excel.

Two independent reviewers extracted data using that form. We extracted data about the following: study identifier; study design; setting; population characteristics; intervention and comparator characteristics; outcomes (quantitative if possible); source of funding and reported conflicts of interests; ethical approval; study limitations or other important comments. For the identified guidance documents, a single reviewer extracted data and a second reviewer verified the extracted data. We extracted data about the publishing organisation and country, whether the documents were dedicated to COVID-19 dead bodies management, as well as specific guidance under each step of the process. We also extracted information on personal protective equipment (PPE).

### Risk of bias assessment

One reviewer was to perform the risk of bias assessment and a second reviewer would verify all assessments. We planned to use the Newcastle-Ottawa scale for non-randomised studies.

### Synthesis

We synthesised the data in both narrative and tabular formats. A single reviewer graded the certainty of the evidence using the GRADE approach and a second reviewer verified all assessments.^6^ When applicable, we followed published guidance for rating the certainty in evidence in the absence of a single estimate of effect.^7^ We present the evidence using GRADE Evidence Profiles developed in the GRADEpro (www.gradepro.org) software.^8 9^

## Results

### Results of the selection process

**Figure 1:**
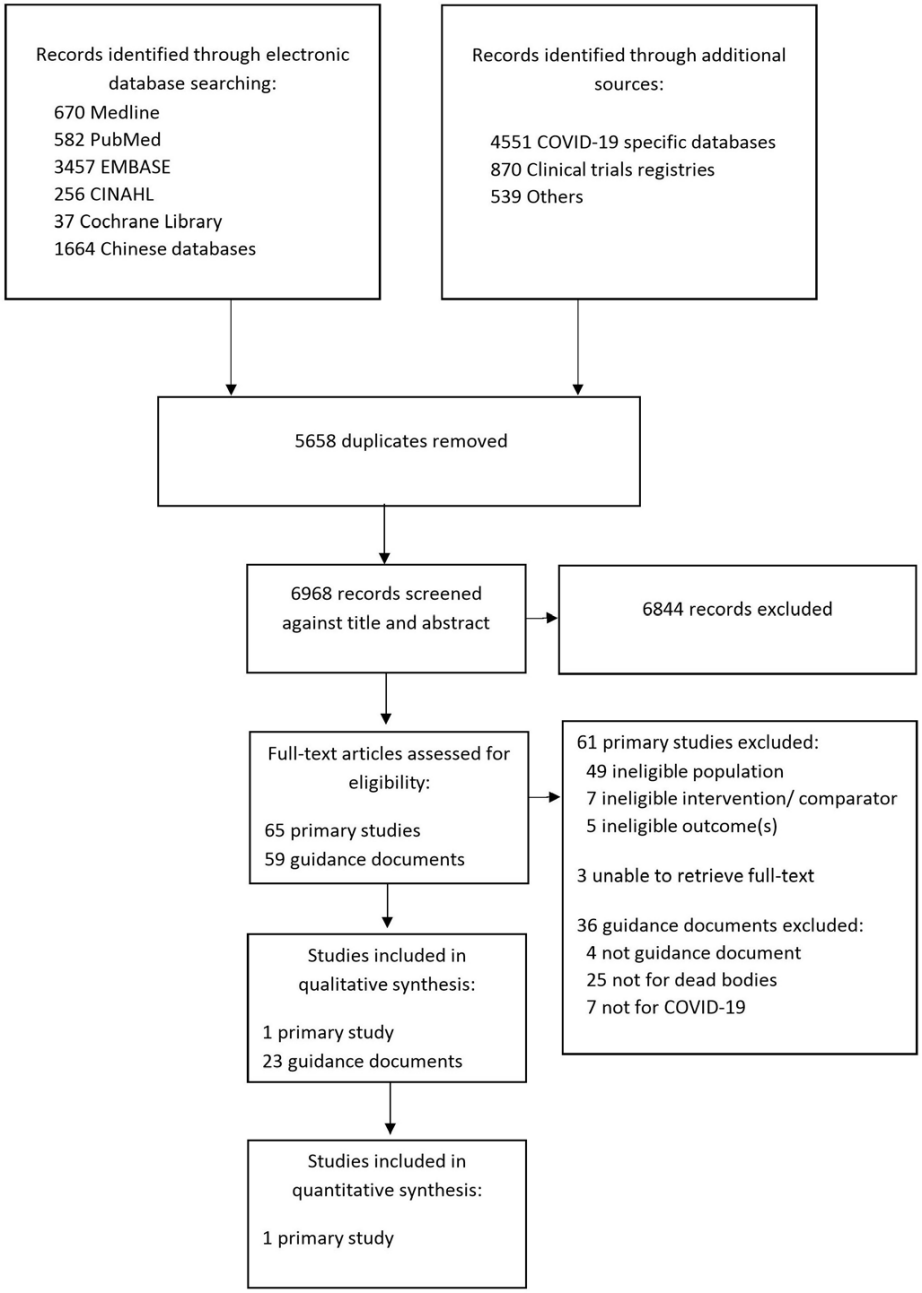
Study selection.

Figure 1 shows the study selection represented in a PRISMA flow chart. We identified 23 guidance documents specific to COVID-19. We did not identify any study providing direct evidence related to COVID-19 (whether on health effects or for contextual evidence). We identified one eligible study that evaluated a biosafety level 3 (BSL-3) laboratory for autopsies of patients with SARS.^10^ We did not identify any relevant systematic review on SARS or MERS.

### Summary of direct empirical findings

Table 1 describes the characteristics of the one included study.^10^ In summary, it was a non-comparative study conducted in a BSL-3 laboratory for autopsies of clinically confirmed or suspected SARS infections in China. It included 16 autopsies performed by 23 pathologists and technicians. The intervention was a complex intervention with components including construction, PPE and disposal. In terms of findings, the authors reported that none of the 23 involved individuals was diagnosed with a SARS infection. In addition, the investigators used the sarin simulant test to assess decontamination. Sarin concentration decreased from 10 to 2 ppm to 0 ppm in the contaminated area and was undetectable in the clean area and the semi-contaminated area.

**Table 1:** Characteristics of the included study

Study	Population	Study type	Setting	Intervention and comparator	Comparator	Outcomes	Risk of bias/quality
Li *et al* (2005)^10^	23 pathologists and technicians performed 16 autopsies	Case study	Autopsy laboratory in China	Multicomponent intervention: construction, PPE, disposal and other measures components	No comparator group	Infections: Proportion of infections: 0/23 Evaluation of decontamination measured by sarin simulant test: in the contaminated area, sarin concentration decreased from 10 to 2 ppm to 0 ppm, and sarin was undetectable in the clean area and the semi-contaminated area	High risk of bias

We report the evidence profile in table 2. We judged the certainty of evidence as very low, mainly due to very serious risk of bias, and very serious imprecision.

**Table 2:** GRADE evidence profile

Certainty assessment							Number of patients		Effect		Certainty	Importance
Number of studies	Study design	Risk of bias	Inconsistency	Indirectness	Imprecision	Other considerations	Specific manage­ment strategy (including ventilation, PPE and disposal)	No specific manage­ment strategy	Relative (95% CI)	Absolute (95% CI)	­	­
Suspected or confirmed COVID-19 infection (assessed with proportion of personnel with SARS infection)												
1	Observational studies	Very serious*	Not serious	Serious†	Very serious‡	None	Out of the 23 personnel who performed the autopsies, none demonstrated any evidence on SARS infection				⨁◯◯◯ Very low	Critical
Suspected or confirmed COVID-19 infection (assessed with evaluation of decontamination measured by sarin simulant test in a SARS laboratory)												
1	Observational studies	Very serious*	Not serious	Very serious§	Not serious	None	Sarin concentration in the contaminated area decreased from 10 to 2 ppm to 0 ppm, and sarin was undetectable in the clean area and the semi-contaminated area				⨁◯◯◯ Very low	Critical

*Downgraded by two levels due to very serious risk of bias. A case study with high risk for confounding bias and selection bias.

†Downgraded by one level due to indirectness, as evidence related to SARS. Refer to the detailed indirectness assessment in the indirectness table.

‡Downgraded by two levels due to very serious imprecision. Low number of participants and events.

§Downgraded by two levels due to very serious indirectness as evidence related to SARS and to a surrogate outcome. Refer to the detailed indirectness assessment in the indirectness table.

PPE, personal protective equipment.

### Summary of the guidance documents

We identified 23 guidance documents providing practical advice on handling suspected or confirmed cases of COVID-19 corpses throughout the different phases. We have summarised the content of these documents in three tables in online supplementary appendix 2.

Table 3 shows the characteristics of the 23 included guidance documents on the strategies for the management of dead bodies of confirmed or suspected COVID-19 cases.

**Table 3:** Characteristics of the guidance documents on the strategies for the management of the bodies of deceased persons with suspected or confirmed COVID-19

Publishing organisation	Country	Month, year of last update	Language	Target audience	Document dedicated to handling deceased	Status of deceased COVID-19	Setting of handling deceased
Centers for Disease Control and Prevention^15^	USA	March, 2020	English	Medical examiners, coroners, pathologists and other workers involved in providing postmortem care, and local and state health departments	✓	Suspected or confirmed	Not specified
Government of India Ministry of Health and Family Welfare Directorate General of Health Services (EMR Division)^13^	India	March, 2020	English	Healthcare workers and personnel who handle dead bodies in isolation area, mortuary, and ambulance and workers in crematorium/burial	✓	Suspected or confirmed	Healthcare facilities/hospitals
European Centre for Disease Prevention and Control^16^	Europe	2020	English	Public health authorities in European Union (EU)/European Economic Area (EAA) Member States and the UK	✓	Suspected or confirmed	Hospitals and communities
Food and Environmental Hygiene Department^17^	Hong Kong	February, 2020	English	Hospitals, public mortuaries, funeral workers and personnel on conveyances	✓	Confirmed	Not specified
Public Health Agency of Sweden^18^	Sweden	March, 2020	Swedish	Healthcare pathological units, forensic units, religious communities and funeral contractors	✓	Confirmed	Not specified
WHO^12^	United Nations	March, 2020	English	Healthcare managers, mortuaries, religious and public health authorities, and families	✓	Suspected or confirmed	Healthcare facilities
Zhejiang University School of Medicine^19^	China	Not specified	English	Medical personnel involved in the management of coronavirus	☓	Suspected or confirmed	Hospital
Ministry of Health in Sri Lanka^20^	Sri Lanka	2020	English	Health sector in Sri Lanka	☓	Suspected or confirmed	Hospitals
European Centre for Disease Prevention and Control^21^	Europe	March, 2020	English	EU/EEA healthcare facilities and healthcare providers	☓	Suspected or confirmed	Hospitals and communities
UK Government^22^	UK	March, 2020	English	First responders and others in close contact with suspected cases including professionals, members of voluntary organisations and emergency service professionals. Also, for Police officers, Border Force officers and Immigration Enforcement officers	☓	Suspected	Communities
Estonian Health Board^23^	Estonia	2020	Estonian	Not specified	✓	Suspected or confirmed	Hospitals
Ministry of Health and Family Welfare, Directorate General of Health Services (Emergency Medical Relief)^14^	Bangladesh	Not specified	English	Healthcare workers and other personnel working in points of entries, quarantine centres, hospitals, laboratories, primary healthcare and community settings	☓	Not specified	Hospitals
Department of Health and Social Care, Public Health Wales, Public Health Agency Northern Ireland, Health Protection Scotland and Public Health England^24^	UK	2020	English	National Health Service (NHS) and healthcare settings (that include infection prevention and control)	☓	Suspected or confirmed	Not specified
The Centre for Respiratory Diseases and Meningitis and Outbreak Response, Division of Public Health Surveillance and Response, National Institute for Communicable Diseases of the National Health Laboratory Services and the National Department of Health, South Africa^25^	South Africa	March, 2020	English	Healthcare workers in medical laboratories, provincial health departments and emergency medical support team	☓	Suspected or confirmed	Ambulance (death during transportation)
Partners in Health^26^	USA	March, 2020	English	Personnel in hospitals involved in screening, triage, infection control and mortuaries	☓	Suspected or confirmed	Hospitals
Clinical Excellence Commission—New South Wales Government^27^	New South Wales	March, 2020	English	Personnel in healthcare or residential and aged care facilities	☓	Suspected or confirmed	Residential and aged care facilities (including multipurpose service residential care)
Clinical Excellence Commission—New South Wales Government^28^	New South Wales	February, 2020	English	Personnel in hospital or similar healthcare setting	☓	Suspected or confirmed	Healthcare facilities/hospitals
The Royal College of Pathologists^29^	UK	February, 2020	English	Pathologists, trainees, anatomical pathology technologists and onsite managers in mortuaries. Also, hospital managers overseeing the mortuary, local authority mortuary managers and coroners	✓	Suspected or confirmed	Hospitals*
Researchers^30^	India	February, 2020	English	Not specified	☓	Not specified	Healthcare facilities
Society of Pathological Doctors, Chinese Medical Doctors Association; Chinese Society of Pathology, Chinese Medical Association^31^	China	March, 2020	Chinese	Pathologists	☓	Suspected or confirmed	Hospitals*
Experts^32^	China	February, 2020	Chinese	Pathologists	☓	Suspected or confirmed	Hospitals*
Syndhedstyrelsen^33^	Denmark	March, 2020	Danish	Danish health authorities, health service settings and healthcare workers	☓	Suspected or confirmed	Not specified
Helsedirektoratet^34^	Norway	March, 2020	Norwegian	Healthcare workers	☓	Suspected or confirmed	Not specified

*Guidance document specific to autopsy.

The majority of the documents were published in English (n=18, 78%). Eight of the 23 documents (35%) were entirely dedicated to handling of COVID-19 dead bodies. Most of the documents specified that bodies were for suspected or confirmed COVID-19 corpses (n=18, 78%). Settings most commonly addressed in those documents were the hospitals (n=12, 52%), healthcare facilities (n=4, 17%) and communities (n=3, 13%).

**Figure 2:**
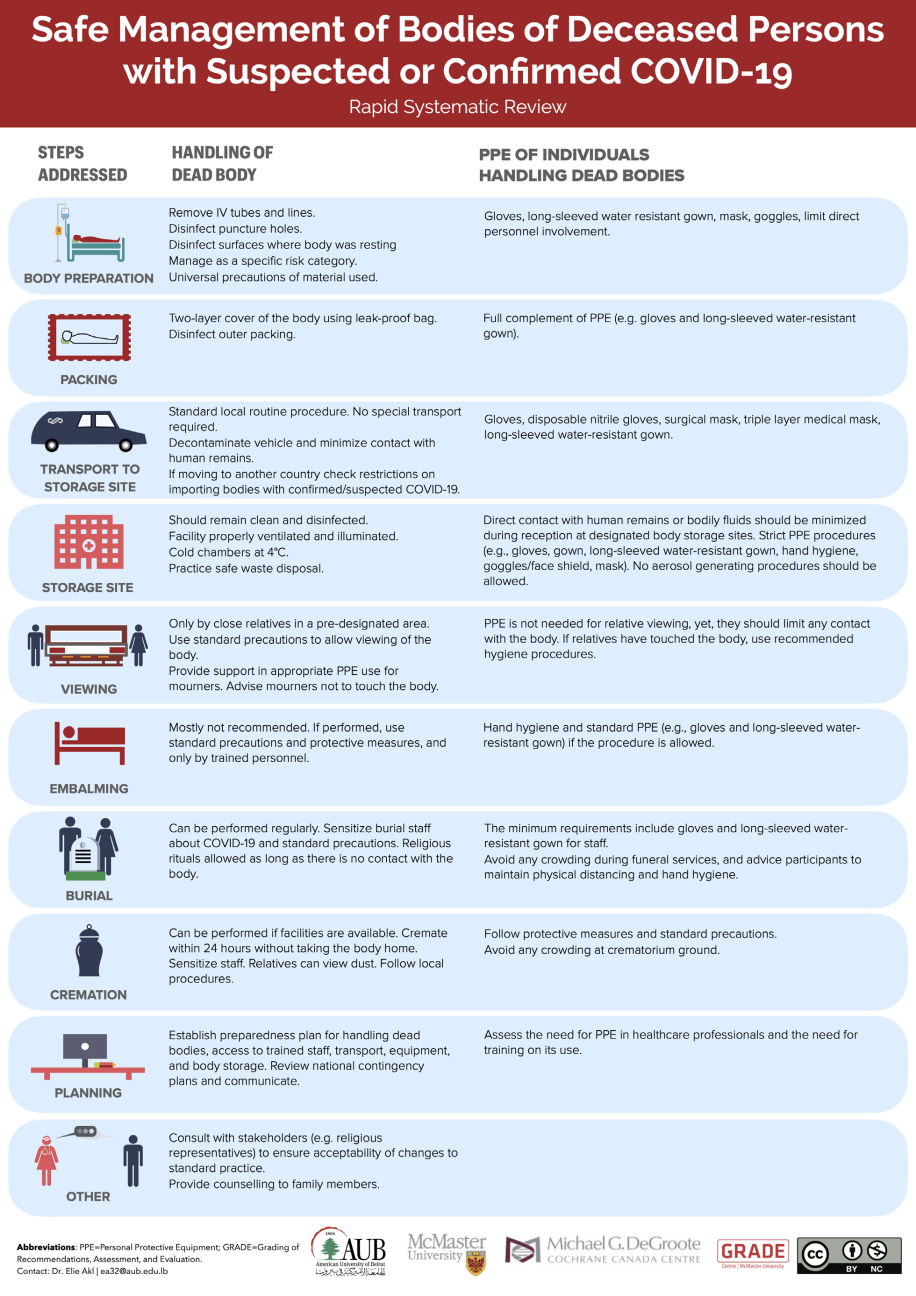
Infographic illustrating the different steps in the management of bodies of deceased person with suspected or confirmed COVID-19. PPE, personal protective equipment.

Online supplementary appendix 2 table A synthesises, across the included guidance documents, the strategies for the management of the bodies of deceased persons with suspected or confirmed COVID-19. The strategies include one or more of the following steps: body preparation (includes healthcare setting and non-healthcare setting), packing, transport to storage site, storage site, viewing, embalming, burial, cremation and other measures. Five documents out of the 23 (22%) report taking into consideration factors related to the context when applying the recommendations provided. These include cultural, religious and familial factors. Out of the five documents, two specify taking these factors into account for all the recommendations, whereas the remaining are specific to cremation (n=2) (online supplementary appendix 2 table A) and autopsies (n=1) (online supplementary appendix 2 table C). Figure 2 represents an infographic summarising the steps reported in the guidance documents. Online supplementary appendix 3 includes the same infogrpahic in the following languages: Arabic, French, German, Italian and Portugese. The expanded version of the infographic is in the online supplementary appendix 1.

Online supplementary appendix 2 table B consists of a matrix indicating what PPE was recommended for use in each of the steps of the management of the bodies of deceased persons with suspected or confirmed COVID-19.

Online supplementary appendix 2 table C provides the recommendations in the guidance documents for performing autopsies on the bodies of deceased persons with suspected or confirmed COVID-19. The main elements are the indications to perform autopsies, the physical environment and disinfection, the professionals involved and limitations on individuals involved.

## Discussion

### Summary of findings

In summary, we did not identify any direct evidence relating to the effects of specific strategies for the management of the bodies of deceased persons with suspected or confirmed COVID-19. Even when expanding to consider indirect evidence, we found scarce literature relating to SARS and MERS infections. The one study we identified provides very low certainty evidence that the specific approach used by Li *et al* to set up the autopsy laboratory reduced the risk of transmission to the personnel handing dead bodies.^10^

### Strengths and limitations

This review has a number of strengths. First, we searched for indirect evidence relating to two other coronaviruses that are similarly dangerous to COVID-19, that is, SARS and MERS. Second, we used a very comprehensive search of both the indexed and grey literatures, with no restrictions to specific languages or study design. Third, and despite the short timeline for conducting the review (7 days), we were able to use a standard systematic review methodology for the primary studies, that is, without any shortcuts sometimes used in rapid reviews. We were able to achieve this through the involvement of a large and experienced systematic review team. One limitation of our study is that we did not use a duplicate approach for data extraction and synthesis for the guidance documents.

### Interpretation of findings

The primary evidence was limited only to management of autopsies and does not cover the main steps of handling of dead bodies. Also, the availability of BSL-3 laboratories and autopsy rooms may be a barrier in many settings, especially for low-resources settings.

The guidance documents proved to be very useful in identifying discrete steps in the management of bodies of deceased persons with suspected or confirmed COVID-19. We noted some variability in the strategies adopted for the different steps across these documents. One limitation of the guidance documents is the lack of consideration of cultural practices around death (eg, cremation vs burial, peri-cremation/burial practices, cultural preferences). Still, and given the lack of primary research, these documents can be helpful to governmental or non-governmental entities when developing such guidance.

### Implications for public health practice

There might be significant harms from the transmission of COVID-19 from the bodies of deceased persons considering the number of deaths currently observed and anticipated in the COVID-19 pandemic. There is one report on a forensic practitioner in Thailand who had contact with biological samples and corpses of COVID-19 and contracted the virus.^11^ This systematic review showed that gaps exist in the evidence base for different management strategies of the bodies of deceased persons for coronaviruses in general, and COVID-19 in particular.

In the absence of such evidence, the synthesis of guidance documents, provided in online supplementary appendix 2, could inform public health protocols around the handling of dead bodies. For example, the current interim WHO guidance draws on evidence from other respiratory viruses including pandemic influenza.^12^ Given gaps in direct evidence on COVID-19, the findings continue to be relevant. However, the interpretation of these findings needs to consider that the included guidance are not supported by direct evidence.

While the risk of infection from dead bodies is deemed to be low, a precautionary approach using PPE including gloves, gown, mask and goggles would be reasonable where direct contact with dead bodies or fluid spray from dead bodies is possible. Only two guidance documents recommended the use of N95 respirators for the handling of dead bodies.^13 14^ Given the absence of direct research evidence, any recommendations on PPE use for handling bodies of deceased people in different settings must balance the uncertainty of the benefits and harms with feasibility considerations, such as PPE stock and availability.

### Implications for research

There are three types of related questions that require research evidence. First, there is a need for evidence on the modes of transmission of COVID-19 from bodies of deceased persons to the different types of individuals handling those bodies. Such evidence is essential to propose potentially effective management strategies. Second, there is a need for evidence on the desirable and undesirable health effects and other consequences of proposed management strategies. Third, there is a need for contextual evidence in relation to these proposed management strategies (ie, acceptability, feasibility, impact on equity, resources considerations). Such evidence is extremely important given the cultural and religious dimensions of the handling of bodies of deceased patients.
