# Supplementary material for: Safe management of bodies of deceased persons with suspected or confirmed COVID-19: a rapid systematic review
Source: BMJ Glob Health. 2020 May 14;5(5):e002650. doi: 10.1136/bmjgh-2020-002650 (PMC7234869; doi:10.1136/bmjgh-2020-002650)
Supplement: Supplementary data [file bmjgh-2020-002650supp001.pdf]

Appendix 1: Search strategies for the different databases ran on March 26, 2020

Medline (OVID)

Ovid MEDLINE(R) and Epub Ahead of Print, In-Process & Other Non-Indexed Citations and Daily 1946 to March 26, 2020

1 (pneumonia/ or pneumonia, viral/ or exp Viruses/) and (exp Disease Outbreaks/ or exp Epidemiology/ or Epidemiology.fs.) (104129)

2 coronaviridae/ or exp coronavirus/ or exp Coronavirus Infections/ or exp Betacoronavirus/ (15998)

3 (Betacoronavirus or Beta-coronavirus or Coronavirus* or COVID).mp. (14380)

4 1 or 2 or 3 (121096)

5 limit 4 to ez="20191101-20200325" (1524)

6 (("2019" adj (novel or new) adj corona*) or ("2019" adj (CoV or nCoV)) or (coronavirus adj (disease adj "2019")) or COVID19 or COVID-19 or ((Novel or New) adj Corona*) or SARS2 or SARS-CoV-2 or (SARS adj2 (coronaviridae or coronavirus)) or ((sars or Coronavirus) adj "2") or nCov or 2019ncov).mp. (4983)

7 5 or 6 (5522)

8 (Cadaver* or Corpse? or dead or remains or carcass* or death* or mortem* or cremat* or Immur* or promessi* or composting or dissolut* or grave? or tomb? or bu?ri* or bur?y* or Adipocere or ((livor or rigor or algor) adj1 mortis) or ((Postmortem or Post-mortem) adj change?) or Cruor or Autolys?s or intermit* or intermis* or interment? or commit* or entombment* or sepltur* or dispos* or deceased or (pass* adj away) or (restless adj bod*)).mp. or exp Cadaver/ (2147642)

9 7 and 8 (670)

PubMed

Search	Query	Items found
#8	Search (#6 AND #7)	582
#7	Search (Cadaver* OR CORpse OR cORpses OR dead OR remains OR carcass* OR death* OR mORtem* OR cremat*[TW] OR Immur* OR promessi OR composting OR dissolut* OR grave OR graves OR tomb OR tombs OR burried OR bury OR burry OR Adipocere OR livOR moRtis* OR rigOR mORtis* OR algOR mORtis* OR PostmoRtem change* OR Post-mORtem change* OR CruOR OR Autolysis OR autolyses OR intermit* OR intermis* OR commit* OR entombment* OR sepltur* OR dispos* OR deceased OR passing away* OR passed away* OR restless bod* OR Cadaver[Mesh])	2195876
#6	Search (#4 OR #5)	4617
#5	Search ((2019-novel-corona* OR 2019-new-corona* OR novel-corona* OR new-corona* OR 2019-Cov OR 2019-nCov OR nCov OR coronavirus disease-2019 OR SARS2 OR SARS-2 OR SARS-CoV-2 OR sars cORona* OR CORonavirus-2 OR 2019ncov))	3907
#4	Search (((#1 OR #2 OR #3) AND 2019/11:2020/03 [crdt]))	2296
#3	Search ((BetacORonavirus[tw] OR Beta-cORonavirus[tw] OR corona[tw] OR corona'[tw] OR corona's[tw] OR OR coronaviral[tw] OR coronavirdae[tw] OR coronavirida[tw] OR coronaviridae[tw] OR coronaviridea[tw] OR coronaviridiae[tw] OR coronavirinae[tw] OR coronavirion[tw] OR coronavirions[tw] OR coronavirologists[tw] OR coronavirology[tw] OR coronaviroses[tw] OR coronavirous[tw] OR coronavirues[tw] OR coronavirus[tw] OR coronavirus'[tw] OR coronavirus's[tw] OR coronaviruscpe[tw] OR coronaviruse[tw] OR coronaviruses[tw] OR coronaviruses'[tw] OR coronaviruslike[tw] OR coronaviser[tw] OR coronaviurs[tw] OR coronaviuses[tw] OR coronavrius[tw] OR coronavvirus[tw] OR COVID[tw]))	21626
#2	Search (((pneumonia[Mesh:noexp] OR pneumonia, viral[Mesh:noexp] OR Viruses[Mesh]) and ("Disease Outbreaks"[Mesh] OR Epidemiology[Mesh] OR Epidemiology [Mesh subject heading])))	30425
#1	Search ((cORonaviridae[Mesh:noexp] OR cORonavirus[Mesh] OR "Coronavirus Infections"[Mesh] OR BetacORonavirus[Mesh]))	16012

EMBASE

No.	Query	Results
#9	#7 AND #8	3457
#8	cadaver*:ti,ab,kw OR corpse$:ti,ab,kw OR dead:ti,ab,kw OR remains:ti,ab,kw OR carcass*:ti,ab,kw OR death*:ti,ab,kw OR mortem*:ti,ab,kw OR cremat*:ti,ab,kw OR immur*:ti,ab,kw OR promessi*:ti,ab,kw OR composting:ti,ab,kw OR dissolut*:ti,ab,kw OR grave$:ti,ab,kw OR tomb$:ti,ab,kw OR bu$ri*:ti,ab,kw OR bur$y*:ti,ab,kw OR adipocere:ti,ab,kw OR livor:ti,ab,kw OR rigor:ti,ab,kw OR ((algor NEAR/1 mortis):ti,ab,kw) OR postmortem:ti,ab,kw OR (('post mortem' NEXT/0 change$):ti,ab,kw) OR cruor:ti,ab,kw OR autolys$s:ti,ab,kw OR intermit*:ti,ab,kw OR intermis*:ti,ab,kw OR interment$:ti,ab,kw OR commit*:ti,ab,kw OR entombment*:ti,ab,kw OR sepltur*:ti,ab,kw OR dispos*:ti,ab,kw OR deceased:ti,ab,kw OR ((pass* NEXT/0 away):ti,ab,kw) OR ((restless NEXT/0 bod*):ti,ab,kw) OR 'cadaver'/exp	2742711
#7	#5 OR #6	26503
#6	((2019 NEXT/0 novel):ti,ab,kw) OR ((2019 NEXT/0 cov):ti,ab,kw) OR ((coronavirus NEXT/0 disease NEXT/0 2019):ti,ab,kw) OR covid19:ti,ab,kw OR 'covid 19':ti,ab,kw OR (((novel OR new) NEXT/0 corona*):ti,ab,kw) OR sars2:ti,ab,kw OR 'sars cov 2':ti,ab,kw OR	18197

	((sars NEAR/2 coronaviridae):ti,ab,kw) OR coronavirus:ti,ab,kw OR sars:ti,ab,kw OR ((coronavirus NEXT/0 '2'):ti,ab,kw) OR ncov:ti,ab,kw OR 2019ncov:ti,ab,kw	
#5	#4 AND [1-11-2019]/sd	9895
#4	#1 OR #2 OR #3	267253
#3	betacoronavirus:ti,ab,kw OR 'beta coronavirus':ti,ab,kw OR coronavirus*:ti,ab,kw OR covid:ti,ab,kw	12845
#2	'coronaviridae'/exp OR 'coronavirus infection'/exp OR 'betacoronavirus'/exp	24506
#1	('pneumonia'/de OR 'virus pneumonia'/de OR 'virus'/exp) AND ('epidemic'/exp OR 'epidemiology'/exp OR epidemiology:lnk)	247225

CINAHL (OVID)

#	Query	Limiters/Expanders	Last Run Via	Results
S10	S6 AND S9	Expanders - Apply equivalent subjects<br>Search modes - Boolean/Phrase	Interface - EBSCOhost Research Databases<br>Search Screen - Advanced Search<br>Database - CINAHL Complete	256
S9	S7 OR S8	Expanders - Apply equivalent subjects<br>Search modes - Boolean/Phrase	Interface - EBSCOhost Research Databases<br>Search Screen - Advanced Search<br>Database - CINAHL Complete	511,164
S8	(MH "Cadaver+")	Expanders - Apply equivalent subjects<br>Search modes - Boolean/Phrase	Interface - EBSCOhost Research Databases<br>Search Screen - Advanced Search<br>Database - CINAHL Complete	11,515
S7	TI ((Cadaver* OR Corpse# OR dead OR remains OR carcass* OR death* OR mortem* OR cremat* OR Immur* OR promessi* OR composting OR dissolut* OR grave# OR tomb# OR bu#ri* OR bur#y* OR Adipocere OR	Expanders - Apply equivalent subjects	Interface - EBSCOhost Research Databases	511,164

	((livor or rigor or algor) N1 mortis) OR ((Postmortem OR Post-mortem) W0 change#) OR Cruor OR Autolys#s OR intermit* OR intermis* OR interment# OR commit* OR entombment* OR sepltur* OR dispos* OR deceased OR (pass* W0 away) OR (restless W0 bod*))) OR AB ((Cadaver* OR Corpse# OR dead OR remains OR carcass* OR death* OR mortem* OR cremat* OR Immur* OR promessi* OR composting OR dissolut* OR grave# OR tomb# OR bu#ri* OR bur#y* OR Adipocere OR ((livor or rigor or algor) N1 mortis) OR ((Postmortem OR Post-mortem) W0 change#) OR Cruor OR Autolys#s OR intermit* OR intermis* OR interment# OR commit* OR entombment* OR sepltur* OR dispos* OR deceased OR (pass* W0 away) OR (restless W0 bod*))) OR MW ((Cadaver* OR Corpse# OR dead OR remains OR carcass* OR death* OR mortem* OR cremat* OR Immur* OR promessi* OR composting OR dissolut* OR grave# OR tomb# OR bu#ri* OR bur#y* OR Adipocere OR ((livor or rigor or algor) N1 mortis) OR ((Postmortem OR Post-mortem) W0 change#) OR Cruor OR Autolys#s OR intermit* OR intermis* OR interment# OR commit* OR entombment* OR sepltur* OR dispos* OR deceased OR (pass* W0 away) OR (restless W0 bod*)))	Search modes - Boolean/Phrase	Search Screen - Advanced Search<br>Database - CINAHL Complete	
S6	S4 OR S5	Expanders - Apply equivalent subjects<br>Search modes - Boolean/Phrase	Interface - EBSCOhost Research Databases<br>Search Screen - Advanced Search<br>Database - CINAHL Complete	1,652
S5	TI (("2019" W0 (novel OR new) W0 corona*) OR ("2019" W0 (CoV OR nCoV)) OR (coronavirus W0 (disease W0 "2019")) OR COVID19 OR COVID-19 OR ((Novel or New) W0 Corona*) OR SARS2 OR SARS-CoV-2 OR (SARS N1 (coronaviridae OR coronavirus)) OR ((sars OR Coronavirus) W0 "2") OR nCov OR 2019ncov) OR AB (("2019" W0 (novel OR new) W0 corona*) OR ("2019" W0 (CoV OR nCoV)) OR (coronavirus W0 (disease W0 "2019")) OR COVID19 OR COVID-19 OR ((Novel or New) W0 Corona*) OR SARS2 OR SARS-CoV-2 OR (SARS N1 (coronaviridae OR coronavirus)) OR ((sars OR Coronavirus) W0 "2") OR nCov OR 2019ncov) OR MW (("2019" W0 (novel OR new) W0 corona*) OR ("2019" W0 (CoV OR nCoV)) OR (coronavirus W0 (disease W0 "2019")) OR COVID19 OR COVID-19 OR ((Novel or New) W0 Corona*) OR SARS2 OR SARS-CoV-2 OR (SARS N1 (coronaviridae OR coronavirus)) OR ((sars OR Coronavirus) W0 "2") OR nCov OR 2019ncov)	Expanders - Apply equivalent subjects<br>Search modes - Boolean/Phrase	Interface - EBSCOhost Research Databases<br>Search Screen - Advanced Search<br>Database - CINAHL Complete	577
S4	S1 OR S2 OR S3	Limiters - Published Date: 20191101-<br>Expanders - Apply equivalent subjects<br>Search modes - Boolean/Phrase	Interface - EBSCOhost Research Databases<br>Search Screen - Advanced Search<br>Database - CINAHL Complete	1,249

S3	TI (Betacoronavirus OR Beta-coronavirus or Coronavirus* OR COVID) OR AB (Betacoronavirus OR Beta-coronavirus or Coronavirus* OR COVID) OR MW (Betacoronavirus OR Beta-coronavirus or Coronavirus* OR COVID)	Expanders - Apply equivalent subjects Search modes - Boolean/Phrase	Interface - EBSCOhost Research Databases Search Screen - Advanced Search Database - CINAHL Complete	1,555
S2	(MH "Coronaviridae+") OR (MH “Coronaviridae Infections+”)	Expanders - Apply equivalent subjects Search modes - Boolean/Phrase	Interface - EBSCOhost Research Databases Search Screen - Advanced Search Database - CINAHL Complete	3,062
S1	((MH "Pneumonia") OR (MH "Pneumonia, Viral") OR (MH "Virus Diseases+") OR ("MH Viruses+")) AND ((MH "Disease Outbreaks") OR (MH "Epidemiology+") OR SU Epidemiology)	Expanders - Apply equivalent subjects Search modes - Boolean/Phrase	Interface - EBSCOhost Research Databases Search Screen - Basic Search Database - CINAHL Complete	77,805

Cochrane Library

ID	Search (Hits)
#1	MeSH descriptor: [Pneumonia, Viral] this term only (51)
#2	MeSH descriptor: [Pneumonia] this term only (1976)
#3	MeSH descripto r: [Viruses] explode all trees (8746)
#4	#1 OR #2 OR #3 (10734)
#5	MeSH descriptor: [Disease Outbreaks] explode all trees (262)
#6	MeSH descriptor: [Epidemiology] explode all trees (37)
#7	(Epidemiology):ti,ab,k (48587)
#8	#5 OR #6 OR #7 (48682)
#9	#4 AND #8 (1315)
#10	MeSH descriptor: [Coronaviridae] this term only (0)
#11	MeSH descriptor: [Coronavirus] explode all trees (11)
#12	MeSH descriptor: [Coronavirus Infections] explode all trees (12)
#13	MeSH descriptor: [Betacoronavirus] explode all trees(10)
#14	(Betacoronavirus or Beta-coronavirus or Coronavirus* or COVID):ti,ab,kw (98)

#15 #9 OR #10 OR #11 OR #12 OR #13 OR #14 with Cochrane Library publication date Between Nov 2019 and Mar 2020 (44)

#16 ((2019 NEXT (novel or new) NEXT corona*)):ti,ab,kw (8)

#17 (("2019" NEXT (CoV or nCoV)) or (coronavirus NEXT (disease NEXT "2019")) or COVID19 or COVID-19 or ((Novel or New) NEXT Corona*) or SARS2 or SARS-CoV-2 or (SARS NEAR/2 (coronaviridae or coronavirus)) or ((sars or Coronavirus) NEXT "2") or nCov or 2019ncov):ti,ab,kw (118)

#18 #15 OR #16 OR #17 (145)

#19 MeSH descriptor: [Cadaver] explode all trees (589)

#20 (Cadaver* or Corpse? or dead or remains or carcass* or death* or mortem* or cremat* or Immur* or promessi* or composting or dissolut* or grave? or tomb? or bu?ri* or bur?y* or Adipocere or ((livor or rigor or algor) NEAR/1 mortis) or ((Postmortem or Post-mortem) NEXT change?) or Cruor or Autolys?s or intermit* or intermis* or interment? or commit* or entombment* or sepltur* or dispos* or deceased or (pass* NEXT away) or (restless NEXT bod*)):ti,ab,kw (139670)

#21 #19 OR #20 (139670)

#22 #18 AND #21 (37)

Appendix 2.

Table A. Synthesis, across the guidance documents, of the strategies for the management of the bodies of deceased persons with suspected or confirmed COVID-19

Steps[1]
**Body preparation (includes healthcare setting and non-healthcare setting)**
Body preparation procedures (healthcare setting)<br>• Remove all tubes, IVs and other lines from the patient [2, 6, 15, 19]<br>• Disinfect any puncture holes or wounds with 1% hypochlorite and dress them with impermeable dressings [2, 19]<br>• If the death occurred in the ICU, the body should be handled by the attending staff, removed from the machines, and placed in a pre-designated area [8]<br>• Plug oral, nasal orifices of a dead body to prevent leakage of body fluids [1,4]; ensure that any body fluids leaking from orifices are contained [6]; clean and disinfect all orifices [19]; use 3,000 mg/L chlorine disinfectant or 0.5% peracetic acid cotton pad or gauze to fill all open channels [21]<br>• Minimal movement and handling of body [6]<br>• Attending physician classifies the dead body into a specific risk category. Tags for categories of dead bodies should be attached to the dead body and the packing (body bag or mortuary sheet) [4]<br>• Exercise caution while handling sharps and dispose them into a sharps' container [2]<br><br>*Cleaning after body preparation (healthcare setting)*<br>• Regular cleaning followed by disinfection [3]<br>• Surfaces where the body was prepared should first be cleaned with soap and water, or a commercially prepared detergent solution [6]<br>• Clean all equipment used (e.g. 70% ethyl alcohol) and the patient's bed (machine washing with warm water at 60−90° C with laundry detergent) [15]<br>• Autoclave or decontaminate used equipment with disinfectant solutions following established infection prevention control practices [2]<br>• Wipe with 1% Sodium Hypochlorite solution all surfaces in the isolation area [floors, bed, railings, side tables, IV stand, etc.], allow a contact time of 30 minutes and air dry [2]<br>• If a surface is likely to be damaged by sodium hypochlorite, use a neutral detergent, followed by a 70% concentration of ethanol [3]<br>• Handle all used/soiled linen with standard precautions and place linen in biohazard bag. Disinfect outer bag surface with hypochlorite solution [2]<br>• After cleaning the surface, place a disinfectant with a minimum concentration of 0.1% (1000 ppm) sodium hypochlorite [bleach], or 70% ethanol for at least 1 minute. Hospital-grade disinfectants may also be used if they have a label claim against emerging viruses, and they remain on surface according to manufacturer's recommendations [6]<br>• In case of shortage in disinfectants, decontaminate with 0.1% sodium hypochlorite after cleaning with a neutral detergent [3]<br>• Treat waste as infectious clinical waste Category B [UN3291] and follow local policies and regulations/ biomedical waste management rules [2, 3, 6]<br>• Cleaning [6] and waste management staff [3, 6] should wear appropriate PPE [6], such as surgical mask, gloves, goggles and gown [3]<br><br>*PPE (healthcare setting)*<br>• Appropriate PPE according to level of interaction with the body [6]; PPE must be removed immediately [19]<br>• Hand hygiene [2, 4, 6, 15, 19]<br>• Gloves [2, 3, 4, 5, 6, 15]<br>• Water resistant gown/ plastic apron [2, 4, 5] over water repellent gown [4], impermeable disposable gown or disposable gown with impermeable apron [6], long-sleeved water-resistant gown [3, 5], gown [6, 15]<br>• Surgical mask [4, 15], medical mask [6], mask [5], N95 mask [2]<br>• Goggles [2, 4, 6, 5, 15] or face shield [4, 6, 5, 15] if there may be splashes [4], eye protection [6]<br>• Limit direct physical involvement of non-healthcare staff when limited PPE availability [3]<br><br>*Body preparation procedures (non-health care setting)*<br>• Minimal contact/exposure [4, 6]<br>• Avoid direct contact with blood or body fluids [4]<br>• Strict personal hygiene [4]<br>• Wounds should be covered with waterproof bandages or dressings [4]<br>• Do not smoke, drink or eat [4]<br>• Do not touch eyes, mouth or nose [4]<br>• Cultural sensitivity [6]

[1] Under each step, precautions apply to the dead body, the person handling the dead body, and/or the physical environment

- Children, adults >60 years old, and anyone with underlying illnesses should not be involved [6]
- A minimum number of people should be involved in preparations [6]; others may observe without touching the body at a minimum distance of 1 meter [6]

Cleaning after body preparation (non-health care setting)

- Cleaning of reusable PPE should be conducted in accordance with manufacturer's instructions for all disinfection products [6]
- Clothing worn to prepare the body should be immediately removed and washed after the procedure, or an apron or gown should be worn [6]
- Belongings of the deceased should be handled with gloves and cleaned with a detergent followed by disinfection with a solution of at least 70% ethanol or 0.1% [1000 ppm] bleach [6]
- Clothing and other fabric belonging to the deceased should be machine washed with warm water at 60−90°C and laundry detergent. If the former is not possible, linens can be soaked in hot water and soap in a large drum using a stick to stir and being careful to avoid splashing. The drum should then be emptied, and the linens soaked in 0.05% chlorine for approximately 30 minutes. Laundry should be rinsed with clean water and linens allowed to dry fully in sunlight [6]

PPE procedures (non-health care setting):

- Disposable gloves [4], gloves [6]
- Water repellent gown [4]
- Apron or gown [6]
- Surgical mask [4], medical mask [activity that may involve splashing] [6]
- Goggles or face shield [activity that may involve splashing] [4, 6]
- Should be used during washing ceremony [23]
- Removal of PPE followed by handwashing with liquid soap and water immediately [4]

Other

- Setting not specified: put used PPE, substrate and bag in a waste bag that is tightly tied back and put in a new bag that is closed. In case of spills with body fluids, dispose waste as contaminated waste [23]
- Unless prior analysis has been performed, analysis should be performed at the ward before ordering transport of the deceased, [11]; analysis of suspected COVID-19 infection should be performed at the Department of Pathology prior to autopsy (analysis is taken from the nasal cavity or pharynx) [11].
- The body shall be transferred by the staff in the isolation ward of the hospital via the contaminated area to the special elevator and out of the ward [7]. Then, disinfect ward and elevator (final disinfection) [7]

Packing

Two layers [2, 4, 7]:

- First layer
  - Robust [4] leak-proof plastic bag [2, 4] that is transparent [4]
  - Recommended thickness not less than 150 μm [4]
  - Double-layer cloth sheet soaked with disinfectant [7]
  - Pins not to be used [4]
- Second layer
  - Double-layer, sealed, leak-proof [7] mortuary sheet [2, 4, 7]
  - Sheet provided by family members [2]
  - Opaque body bag [4]

Other packing [8, 14, 16, 17]

- Sealed body bag [8, 17]
- Leak proof [14, 16] double body bag [14]
- Layered corpse bag [21]
- Wrap body in cloth and transfer as soon as possible to mortuary [6]
- In the mortuary, the body should be placed in disposable plastic bags if soiled with blood or body fluids [19]
- No requirement for a body bag [6, 13], although it may be used for other reasons [e.g. excessive body fluid leakage] [6]
- No need to disinfect body before transfer to mortuary [6]

Disinfection of the outer packing

- Disinfect with 1% hypochlorite [2]
- Wipe with 1 in 4 diluted household bleach [mixing 1 part of 5.25% bleach with 4 parts of water] and allow to air dry [4]
- Soak with chlorine containing disinfectant [7]
- Wipe with 0.05% chlorine solution [14]
- Disinfect with a product that contains EPA-approved emerging viral pathogens claims expected to be effective against COVID-19 applied according to the manufacturer's recommendations [1]

PPE

- Gloves [3] and long-sleeved water-resistant gown [3]
- Full complement of PPE (goggles, face-shield, mask, gloves, coverall/gowns (with or without aprons), head cover and shoe cover) (packing classified as high risk procedure) [12]

Transport/transfer

- Follow standard routine procedures [1], local procedures [5], the procedures laid down by the hospital [11]
- No special transport equipment or vehicle is required [6]
- Transport by special car [21]
- Decontaminate vehicle after transfer with 1% Sodium Hypochlorite [2]
- (a) when there are no visible pollutants, spray with 1000 mg/L of chlorine-containing disinfectant or 500mg / L of chlorine dioxide disinfectant to wet the surface of objects in the vehicle for 30 min; (b) when there are visible pollutants, use disposable absorb material with 5000 – 10000 mg/L chlorine-containing disinfectant solution (or a disinfectant that can reach a high level of disinfection wipes/dry wipes) to remove pollutants, and then follow the procedures under (a) [21]
- Minimize direct contact with human remains or bodily fluids [3]
- Coffin should be preferably sealed [8]
- Inform transporting staff of the potential risk of infection [11]
- Transfer outside the country (specific to Denmark): no restrictions on the export of corpses, but anyone exporting a corpse abroad must be aware of whether the country in question has restrictions on the import of corpses of the deceased with COVID-19 [22]

PPE

- Gloves [2, 3, 12], disposable nitrile gloves [1]
- Surgical mask [2], triple layer medical mask [12]
- Long-sleeved water-resistant gown [3]
- PPE not otherwise specified [11]
- Used PPE considered as infectious waste [11]
- Transport to mortuary classified as low risk procedure [12]

Storage site

Physical environment

- Should remain clean [2, 6, 23], daily cleaning [15]
- Clean with sodium hypochlorite 1% solution for chamber door, handles and floor after removing the body [2, 23]; chlorine 1% or bleach 5% [15]; 1% Hypochlorite solution for environmental surfaces, instruments and transport trolleys [2, 23]
- Proper ventilation [6]
- Adequate lightening [6]
- Store in cold chambers maintained at 4°C [2, 23], -4°C [19]
- Perform safe disposal of waste [23]

PPE

- Minimize direct contact with human remains or bodily fluids during reception at body storage sites [3]
- Strict PPE procedures [19]
- Gloves [2, 3, 6, 12, 15], gown [6, 15], long-sleeved water-resistant gown [3], according to level of interaction with the body [6]
- Hand hygiene [6, 23], before and after interaction with body and environment [6]
- Goggles/face shield [6, 15]
- Medical mask [2, 6], surgical mask [15], N95 mask [12]
- No aerosol generating procedures should be allowed [12]
- If suspected case: stored in the hospital refrigerator until a response is obtained [11]

Viewing

Hospital setting

- Viewing is permitted [13]; viewing of the body only by close relatives is allowed in a pre-designated area in the hospital [8]
- Use of standard precautions by the staff to view the body by unzipping the face end of the bag [2]; refrain from touching the body [16]
- Use standard precautions and PPE (gloves and long-sleeved water-resistant gown) if mourners/religious representatives are to touch the body [3]
- Staff needs to ensure that mourners receive support in appropriate PPE use [3]; give the family clear instructions not to touch or kiss the body [6]
- In case of limited PPE availability or shortage of staff to supervise visitors, consider restricting touching [3]

- Apply standard precautions including hand hygiene if family wishes only to view the body [6, 23] without touching it [6]

During funeral [4], burial by family members, or for deaths at home [6]:

- Viewing is allowed [4, 6], without touching or kissing, and with hand hygiene and maintaining distance [6]

Other/setting not specified:

- PPE needed for relative viewing [23]
- Relatives should limit contact with the dead [23]
- If the relatives have touched the dead, they need to perform hand hygiene before touching anything else in the room [23]
- Body should not be viewed after sealing [8]

Embalming

- Not allowed/not recommended [2, 4, 6, 8, 12]
- Permitted with the following recommendations [3,5]:
  - Using standard precautions and PPE (gloves and long-sleeved water-resistant gown) [3]
  - Using protective measures including basic hygiene practices and protection from contamination [5]
- Performed by trained autopsy techniques according to local procedures [5]
- Permitted [13]

Burial

- Can be performed regularly [3]
- Shrouding and preparation of the body for viewing and/or funeral can be performed by using appropriate standard precautions and PPE (minimum requirements include gloves and long-sleeved water-resistant gown) [3]
- Sensitize burial staff that COVID-19 does not pose additional risk [2]
- Religious rituals such as reading from religious scripts, sprinkling holy water and any other last rites that does not require touching of the body can be allowed [2]
- Bathing, kissing, hugging of the dead body should not be allowed [2]
- Staff should practice standard precautions of hand hygiene and use of masks and gloves [2]
- Burial staff and family members should perform hand hygiene after burial [2]
- Hygienic preparation in funeral parlour is allowed with PPE (disposable gloves, water repellent gown and surgical mask) [4]
- Burial is performed without any further action [5]
- Individuals placing the body in grave, on funeral pyre, etc., should wear gloves and wash hands with soap and water after removal of gloves after burial [6]
- In cases where the burial practice is not to use the coffin, the body should be wrapped in burial sheet/textiles and then placed in a closed bag [23]
- Abide by national and local requirements that may dictate the handling and disposition of remains [6]

Crowding

- Avoid large gathering [2] and limit number of participants [6] at burial ground as close family may be symptomatic and/or shedding the virus [2]
- Other funeral ceremonies should be postponed to the most possible [6]
- Participants should practice physical distancing, respiratory etiquette and hand hygiene [6]

Cremation

- Can be performed regularly [3]; performed where needed facilities are available [5]
- Advisable [4]
- Perform within 24 hours of death without taking home [8]
- Sensitize crematorium staff that COVID-19 does not pose additional risk [2]
- Ash does not pose any risk and can be collected to perform the last rites [2]
- Relatives can view dust [5]
- Follow protective measures (e.g. basic hygiene practices and protection from contamination) [5]; staff should practice standard precautions of hand hygiene and use of masks and gloves [2]. Family members should perform hand hygiene after cremation [2]
- Cremation should be under the supervision of Ministry of Health (MoH)/ public health inspectors (PHI) and area police [8]
- Abide by national and local requirements that may dictate the handling and disposition of remains [6]
- Take into consideration the feasibility, acceptability and availability of resources with respect to familial, cultural, and/or religious factors [6,14]

Crowding<br><br>Avoid large gathering at crematorium ground as close family may be symptomatic and/or shedding the virus [2]
**Planning**
• Establish preparedness plan for handling dead bodies. Plan should address access to trained staff, transportation, equipment and physical structures needed for the storage of bodies and performance of burials and cremations [3]<br>• In case the current capacity to manage dead bodies is exceeded, verify current applicability of national civil contingency plans for surge capacity to manage dead bodies [3]<br>• Authorities should manage each situation on a case-by-case basis, balancing rights of family, need to investigate cause of death, and risks of exposure to infection [6]<br>• Hospitals should be prepared to manage an increased number of dead bodies [9]<br><br>*Considering other factors*<br><br>• Consult with stakeholders and societal leaders, particularly religious representatives, to ensure acceptability of changes to standard practice as they may vary according to local, cultural and religious context [3]<br>• Adapt the recommendations to take into account cultural and religious concerns [4]
**Other**
• Identify key professional groups that are involved in handling dead bodies within and outside the healthcare system (e.g. healthcare staff, primary care, morgue staff, funeral agencies, transportation services, religious representatives, and organizational structures undertaking burials or cremations). Assess need of PPE for each group and train on PPE use [3]<br>• Provide counseling to family members [2, 15]

Table B. Personal protective equipment (PPE) the guidance documents recommend by the steps of the management of the bodies of deceased persons with suspected or confirmed COVID-19 (n=23)

Step	Hand hygiene n (%)	Gloves n (%)	Medical/ surgical mask n (%)	Respirator (N95 or similar) n (%)	Long-sleeved gown n (%)	Apron n (%)	Goggles/ face shield n (%)
Body preparation	5 (22) [2,4,6,15,19]	6 (26) [2-6, 15]	4 (17) [4-6, 15]	1 (4) [2]	5 (22) [3-6,15]	3 (13) [2,4,5]	5 (22) [2,4,5,6,15]
Packing	0	2 (9) [3,12]	1 (4) [12]	0	2 (9) [3,12]	1 (4) [12]	1 (4) [12]
Transport/transfer to storage site	0	4 (17) [1-3,12]	2 (9) [2,12]	0	1 (4) [3]	0	0
Storage site	2 (9) [6,23]	5 (22) [2,3,6,15,12]	3 (13) [2,6,15]	1 (4) [12]	3 (13) [3,6,15]	0	2 (9) [6,15]
Viewing	2 (9) [6,23]	2 (9) [2,3]	1 (4) [2]	0	1 (4) [3]	0	0
Embalming	0	1 (4) [3]	0	0	1 (4) [3]	0	0
Burial	2 (9) [2,6]	4 (17) [2-4,6]	2 (9) [2,4]	0	2 (9) [3,4]	0	0
Cremation	1 (4) [2]	1 (4) [2]	1 (4) [2]	0	0	0	0

Table C. Recommendations in guidance documents for performing autopsies on the bodies of deceased persons with suspected or confirmed COVID-19 infection (n=15)

Recommendations	n (%)	References
**Preferences in regard to performing autopsies**		
– not recommended	2 (13)	[2, 4]
– recommended only if necessary‡	6 (40)	[1, 3, 6, 8, 11, 12]
– should be consistent with local procedures	3 (20)	[5, 11, 18]
– main reason to perform is a suspected coronavirus infection	4 (27)	[8, 11, 18, 20]
**Physical environment**		
Characteristics of physical environment		
– require negative pressure room*	5 (33)	[1, 2, 8, 20, 21]
– make available biosafety containers for all specimens	2 (13)	[1, 18]
– divide areas into contaminated area, potentially contaminated area, buffer zones, and safety zone	2 (13)	[20, 21]
Precautionary measures		
– reduce aerosol generation or consider additional respiratory protection	6 (40)	[1-3, 6, 8, 18]
– use hand shears, round-ended scissors or oscillator saw with vacuum	4 (27)	[1, 2, 8, 18]
– hold firmly unfixed organs and slice with a sponge	2 (13)	[2, 18]
– allow only one personnel to cut at a time	3 (20)	[1, 2, 18]
– use caution when handling sharps (e.g., use PM40 blades with blunted points)	2 (13)	[1, 20]
– avoid cross-contamination and maintain hand hygiene	1 (7)	[23]
– keep a logbook to record all activities (including cleaning)	1 (7)	[1]
– provide training and demonstration of safety practices	1 (7)	[18]
Cleaning		
– keep ventilation systems active	3 (20)	[1, 6, 18]
– apply disinfectants as instructed and use body bags**	6 (40)	[1, 2, 6, 8, 20, 21]
– ensure adequate contact time	1 (7)	[1]
– avoid using cleaning methods that generate aerosols	1 (7)	[1]
– use absorbent materials to collect gross contamination and liquids	1 (7)	[1]
– disinfect or autoclave non-disposable instruments	2 (13)	[1, 6]
wash reusable items with detergents on warmest setting, rinse with water, disinfect, and then dry	1 (7)	[1]
Waste disposal		
– dispose PPE and medical waste based on local procedures	1 (7)	[1]
– dispose human tissues based on routine procedures for pathological waste	1 (7)	[1]
– use leak-proof biohazard bag to remove materials or clothing	2 (13)	[1, 21]
– place sharps in puncture-proof labeled containers	3 (20)	[1, 2, 18]
**Personal Protective Equipment (PPE)**		
– general recommendation on using PPEs and safety measures	11 (73)	[1-6, 8, 9, 12, 18, 23]
– disposable nitrile gloves	8 (53)	[1, 5, 6, 12, 18, 20, 21, 23]
– heavy-duty gloves over nitrile gloves (risk of injuries)	4 (27)	[1, 5, 6, 18]
– long-sleeved impermeable gown and/or impermeable apron	6 (40)	[1, 2, 5, 6, 18, 23]
– face shield/mask and/or goggles	7 (47)	[1,2,5,6,18,20,23]
– N-95 respirator or equivalent	4 (27)	[1, 2, 6, 18]
– powered, air-purifying respirators (risk of aerosol-generation)	4 (27)	[1, 5, 18, 20]
– head and shoe cover	3 (20)	[2, 6, 18]
**Types of personnel involved**		
– pathologist	3 (20)	[4, 8, 21]
– senior-most Judicial Medical Officer with senior morgue attendants	1 (7)	[8]
– circulator assistant and anatomical pathology technologists (optional)	1 (7)	[18]
– pathology trainees after having demonstrated knowledge of safe protection practices under supervision of senior staff	1 (7)	[18]

**Restrictions on personnel involvement**		
– limit number of personnel involved	6 (40)	[1, 2, 4, 6, 20, 21]
– limit number of forensic experts involved	1 (7)	[2]

ǂ recommends taking into consideration family and cultural wishes [1]
* not required if collecting only nasopharyngeal (NP) swab [1]
** disinfect the body and exterior of body bag with 1% sodium hypochlorite after procedure [2]

Appendix 3. Infographic summarising the steps reported in the guidance documents translated to Arabic, French, German, Italian and Portugese.

الإدارة الآمنة لجثث المتوفين المصابين المشتبه أو المؤكدة إصابتهم بفيروس كورونا الجديد

مراجعة منهجية سريعة للدراسات لصالح منظمة الصحة العالمية

الخطوات الموجهة	التعامل مع الجثث	معدات الوقاية الشخصية للأفراد الذين يتعاملون مع الجثث
تحضير الجثة	قم بإزالة الأنابيب والخطوط الوريدية<br>طهر ثقوب الحقن<br>طهر الأسطح حيث كان جثة المتوفي تعامل معها تحت فئة مخاطر محددة الاحتياطات العالمية للمواد المستخدمة	قفازات، ثوب طويل الأكمام مقاوم للماء، قناع، نظارات واقية، الحد مشاركة الأشخاص المباشرة
التعبئة	غطاء من طبقتين للجسم باستخدام حقيبة مانعة للتسرب<br>طهر التغليف الخارجي	عدة كاملة من معدات الحماية الشخصية مثل القفازات وثوب طويل الأكمام مقاوم للماء
النقل إلى موقع التخزين	الإجراء الروتيني المحلي القياسي<br>لا يوجد نقل خاص مطلوب<br>تطهير السيارة وتقليل الاتصال مع الرفات البشرية<br>في حالة الانتقال إلى دولة أخرى ، تحقق من القيود المفروضة من الدولة على استقبال جثث المشتبه أوالمؤكد إصابتهم بفيروس كورونا الجديد	قفازات، قفازات النتريل قابلة للتخلص منها، قناع جراحي، قناع طبي ثلاثي الطبقات، ثوب طويل الأكمام مقاوم للماء
موقع التخزين	يجب أن تظل نظيفة ومعقمة<br>مكان جيد التهوية وإضاءة<br>غرف مبردة عند 4 درجات مئوية<br>تدرب على التخلص الآمن من النفايات	يجب التقليل من المخالطة المباشرة بالرفات البشرية أوسوائل الجسم أثناء الاستقبال في المواقع المخصصة لتخزين الجثة. إجراءات صارمة على معدات الوقاية الشخصية على سبيل المثال، القفازات، ثوب، ثوب طويل الأكمام مقاوم للماء، نظافة اليد، نظارات/درع الوجه، قناع<br>يجب عدم السماح بإجراءات توليد الهباء الجوي
المشاهدة	فقط من قبل الأقارب المقربين في منطقة محددة مسبقًا<br>استخدم الاحتياطات القياسية للسماح بعرض جثة المتوفي<br>قدم الدعم في استخدام معدات الوقاية الشخصية المناسبة للمشيعين<br>انصح المعزين بعدم لمس جثة المتوفي	لا حاجة إلى معدات الحماية الشخصية في العرض للأقارب، ومع ذلك، يجب الحد من أي اتصال بالجثة<br>إذا لامس الأقارب الجثة، فاستخدم إجراءات النظافة الموصى بها
تحنيط	لا ينصح في الغالب<br>إذا تم تنفيذ ذلك، استخدم الاحتياطات القياسية وتدابير الحماية وفقط من قبل الموظفين المدربين	نظافة اليدين ومعدات الوقاية الشخصية القياسية على سبيل المثال، قفازات وثوب طويل الأكمام مقاوم للماء إذا كان الإجراء مسموحًا به
دفن	يمكن إجراؤها بانتظام<br>توعية موظفي الدفن حول الاحتياطات القياسية وفيروس كورونا الجديد<br>الطقوس الدينية مسموح بها طالما لا يوجد اتصال مع الجثة	الحد الأدنى من المتطلبات يشمل القفازات وثوب طويل الأكمام مقاوم للماء للموظفين
حرق الجثث	يمكن القيام به إذا كانت الإمكانيات متوفرة<br>حرق الجثث في غضون 24 ساعة دون أخذ الجثة إلى المنزل<br>توعية الموظفين<br>يمكن للأقارب مشاهدة الرماد<br>اتبع الإجراءات المحلية	تجنب أي ازدحام أثناء خدمات الجنازة، ونصح المشاركين بالحفاظ على التباعد الجسدي ونظافة اليدين<br>اتبع الإجراءات الوقائية والاحتياطات القياسية<br>تجنب الازدحام في مكان حرق الجثث
التخطيط	وضع خطة استعداد للتعامل مع الجثث، والتواصل مع الموظفين المدربين، ووسائل النقل، والمعدات، وتخزين الجثة التواصل ومراجعة خطط الطوارئ الوطنية	تقييم الحاجة إلى معدات الوقاية الشخصية للمتخصصين في الرعاية الصحية والحاجة إلى التدريب على استخدامها
آخر	تشاور مع أصحاب الشأن (مثل رجال الدين) لضمان قبول التغييرات على الممارسة التقليدية<br>تقديم المشورة لأفراد الأسرة	

الاختصارات: GRADE=Grading of Recommendations, Assessment, and Evaluation

للتواصل: Dr Elie Akl | ea32@aub.edu.lb

CC BY NC

Gestion adéquate des corps de personnes décédées avec virus COVID-19 suspecté ou confirmé

Une revue systématique rapide pour l'Organisation Mondiale de la Santé

ÉTAPES ABORDEES	GESTION DES CADAVRES	EPI DE PERSONNES MANIPULANT LES CADAVRES
PREPARATION DU CORPS	Retirer les tubes et les lignes IV.<br>Désinfecter les trous de ponction.<br>Désinfecter les surfaces sur lesquelles le corps reposait.<br>Gérer comme une catégorie de risque spécifique.	Gants, blouse à manches longues résistante à l'eau, masque, lunettes ; limiter l'implication directe du personnel.
EMBALLAGE	Couverture du corps en deux couches à l'aide d'un sac étanche.<br>Désinfecter l'emballage extérieur.	Complément total d'EPI (par exemple, gants et blouse à manches longues résistante à l'eau).
TRANSPORT VERS LE SITE D'ENTREPOSAGE	Procédure de routine locale standard. Pas de transport spécial requis.<br>Décontaminer le véhicule et minimiser le contact avec les restes humains.<br>En cas de déplacement dans un autre pays, vérifier les restrictions sur l'importation de corps avec virus COVID-19 confirmé/ suspecté.	Gants, gants en nitrile jetables, masque chirurgical, masque médical triple couche, blouse à manches longues résistante à l'eau.
SITE D'ENTREPOSAGE	Doit rester propre et désinfecté.<br>Établissement correctement ventilé et éclairé.<br>Chambres froides à 4°C.<br>Pratiquer l'élimination adéquate des déchets.	Le contact direct avec les restes humains ou les fluides corporels doit être réduit au minimum lors de la réception dans les sites désignés d'entreposage des corps. Procédures strictes d'EPI (par exemple, gants, blouse, blouse à manches longues résistante à l'eau, hygiène des mains, lunettes de protection/ écran facial, masque). Aucune procédure génératrice d'aérosols ne devrait être autorisée.
VISUALISATION	Uniquement par des parents proches dans une zone prédéfinie.<br>Prendre des mesures de protection standard pour permettre la visualisation du corps.<br>Aider les personnes en deuil avec l'utilisation appropriée des EPI.<br>Conseiller aux personnes en deuil de ne pas toucher le corps.	Les EPI ne sont pas nécessaires pour une visualisation de la part des proches. Cependant, ils devraient limiter tout contact avec la dépouille. En cas de contact avec le corps de la part des proches, utilisation des procédures d'hygiène recommandées.
EMBAUMEMENT	Généralement non recommandé. Si effectué, utiliser des précautions et des mesures de protection standard, et uniquement par un personnel formé.	Hygiène des mains et EPI standard (par exemple, gants et blouse à manches longues résistante à l'eau) si la procédure est autorisée.
ENTERREMENT	Peut être effectué normalement. Sensibiliser le personnel de l'enterrement au COVID-19 et aux précautions standard. Les rituels religieux sont autorisés tant qu'il n'y a pas de contact avec corps.	Les exigences minimales pour le personnel comprennent des gants et une blouse à manches longues résistante à l'eau.<br>Éviter tout encombrement lors des funérailles et conseiller aux participants de maintenir la distanciation physique et l'hygiène des mains.
INCINERATION	Peut être effectuée si des installations sont disponibles. Incinérer dans les 24 heures sans ramener le corps à la maison. Sensibiliser le personnel. Les proches peuvent voir la poussière. Suivre les procédures locales.	Suivre les mesures de protection et les précautions standard.<br>Éviter tout encombrement au lieu d'incinération.
PLANIFICATION	Établir un plan de préparation pour la manipulation des cadavres, l'accès à un personnel qualifié, le transport, l'équipement et l'entreposage des corps. Réviser les plans d'urgence nationaux et les communiquer.	Évaluer le besoin d'EPI chez les professionnels de santé et le besoin de formation à son utilisation.
AUTRE	Consulter les parties prenantes (par exemple, les représentants religieux) pour garantir l'acceptabilité des changements apportés aux pratiques standard.<br>Fournir des conseils aux membres de la famille.	

Abréviations: EPI = équipement de protection individuelle; GRADE=Grading of Recommendations, Assessment, and Evaluation.

Contact: Dr Elie Akl | ea32@aub.edu.lb

Sicherer Umgang mit den Körpern von verstorbenen Personen mit Verdacht auf eine COVID-19-Infektion oder einer bestätigten COVID-19-Infektion

Eine systematische Übersichtsarbeit für die Weltgesundheitsorganisation

ANGESPROCHENE SCHRITTE	UMGANG MIT DEM LEICHNAM	PERSÖNLICHE SCHUTZAUSRÜSTUNG FÜR PERSONEN DIE MIT LEICHEN UMGEHEN
VORBEREITUNG DES LEICHNAMS	Intravenöse und andere Zugänge entfernen.<br>Punktionsstellen desinfizieren.<br>Flächen, auf denen der Körper lag, desinfizieren.<br>Verhalten wie bei einer spezifischen Risikokategorie.<br>Universelle Vorsichtsmaßnahmen für das verwendete Material.	Handschuhe, langärmeliger, wasserdichter Kittel, Maske, Schutzbrille, Begrenzung/Minimierung des Personals mit Kontakt.
ABDECKUNG	Zweilagiges Abdecken des Leichnams mit auslaufsicherem Beutel.<br>Desinfektion der äußeren Abdeckung.	Vollständiges Auffüllen der persönlicher Schutzausrüstung (z.B. Handschuhe und langärmelige, wasserfeste Kittel).
TRANSPORT ZUR AUFEBWAHRUNGSSTELLE	Lokale Standard-Routineverfahren verwenden. Kein Sondertransport erforderlich.<br>Fahrzeug dekontaminieren und Kontakt mit Leichnam minimieren. Bei Transport in ein anderes Land Einschränkungen für die Einfuhr von Leichen mit bestätigtem/vermutetem COVID-19 überprüfen und berücksichtigen.	Handschuhe, Einweghandschuhe aus Nitril, chirurgische Maske, dreilagige medizinische Maske, langärmeliger, wasserdichter Kittel.
AUFEBWAHRUNGSSTELLE	Sollte stets sauber und desinfiziert bleiben.<br>Die Einrichtung sollte ordnungsgemäß belüftet und beleuchtet sein.<br>Kühlkammern bei 4°C.<br>Sichere Abfallentsorgung praktizieren.	Beim der Übergabe an ausgewiesene Aufbewahrungsstelle direkten Kontakt mit menschlichen Überresten oder Körperflüssigkeiten minimieren. Strenge Nutzung von persönlicher Schutzausrüstung und Anwendung von Schutzmaßnahmen (z.B. Handschuhe, Kittel, langärmeliger, wasserdichter Kittel, Handhygiene, Schutzbrille/Gesichtsschutz, Maske). Keine Nutzung aerosolerzeugenden Verfahren.
BETRACHTUNG DES LEICHNAMS	Nur durch nahe Verwandten in einem vorher festgelegten Bereich. Standard-Vorsichtsmaßnahmen verwenden, um die Betrachtung des Leichnams zu ermöglichen.<br>Trauernden Unterstützung bei der angemessenen Verwendung von persönlicher Schutzausrüstung anbieten. Den Trauernden raten, den Leichnam nicht zu berühren.	Persönliche Schutzausrüstung ist für die Betrachtung des Leichnams nicht erforderlich, dennoch sollte jeder Kontakt mit dem Leichnam eingeschränkt werden. Wenn Angehörige den Leichnam berührt haben, empfehlen wir die Anwendung üblicher Hygieneverfahren.
EINBALSAMIERUNG	Meistens nicht empfohlen. Falls durchgeführt, Standard-Vorsichts- und Schutzmaßnahmen verwenden. Nur durch geschultes Personal.	Handhygiene und Persönliche Schutzausrüstung (z.B. Handschuhe und langärmelige, wasserfeste Kittel), wenn das Verfahren zulässig ist.
BEERDIGUNG	Kann regelmäßig durchgeführt werden. Sensibilisieren des Bestattungspersonals für COVID-19 und die Standard-Vorsichtsmaßnahmen. Religiöse Rituale sind erlaubt, solange es keinen Kontakt mit dem Leichnam gibt.	Die Mindestanforderungen umfassen Handschuhe und langärmelige, wasserfeste Kittel für das Personal.<br>Vermeidung von Menschenmengen während der Beerdigung und Empfehlung physische Distanzierung und Handhygiene einzuhalten.
EINÄSCHERUNG	Kann durchgeführt werden, wenn Einrichtungen hierfür vorhanden und zugänglich sind. Einäscherung innerhalb von 24 Stunden, ohne den Leichnam vorher nach Hause zu bringen.<br>Personal sensibilisieren. Angehörige dürfen Asche sehen.<br>Befolgen der örtlichen Abläufe.	Schutzmaßnahmen und Standardvorkehrungen befolgen.<br>Vermeidung von Menschenmengen auf dem Gelände de Krematoriums.
PLANUNG	Erstellung eines Versorgungsplans für den Umgang mit Leichen, Zugang zu geschultem Personal, Transport, Ausrüstung und Lagerung von Leichen. Überprüfung und Kommunikation der nationalen Notfallpläne.	Beurteilung des Bedarfs an persönlicher Schutzausrüstung und Schulungen zur Nutzung von persönlicher Schutzausrüstung durch Mitarbeiter der Gesundheitsberufe.
ANDERE	Konsultation von Interessengruppen (z.B. religiösen Vertretern), um die Akzeptanz von Änderungen der Standardverfahren zu gewährleisten.<br>Beratung von Familienangehörigen anbieten.	

Abkürzungen: GRADE=Grading of Recommendations, Assessment, and Evaluation.
Kontakt: Dr Elie Akl | ea32@aub.edu.lb

Trattamento controllato delle salme di pazienti deceduti per COVID-19 sospetta o confermata

Una Rapida Revisione Sistematica effettuata per conto della Organizzazione Mondiale della Sanità

PROCEDURE AFFRONTATE	TRATTAMENTO DELLE SALME	DISPOSITIVI DI PROTEZIONE INDIVIDUALE PER IL PERSONALE ADDETTO AL TRATTAMENTO DELLE SALME
PREPARAZIONE DELLE SPOGLIE	Rimozione dei cateteri e degli accessi venosi.<br>Disinfezione dei fori di accesso.<br>Disinfezione dei siti di deposizione della salma.<br>Il trattamento della salma come specifica categoria di rischio.<br>Precauzioni universali per quanto riguarda il materiale adoperato.	I guanti, i camici impermeabili a maniche lunghe, le maschere e gli occhiali protettivi limitano l'esposizione diretta da parte del personale.
DEPOSIZIONE DEL CORPO NELLA SACCA MORTUARIA	Assicurare una doppia copertura per la salma tramite una sacca mortuaria a tenuta ermetica.<br>Disinfezione della superficie esterna della sacca mortuaria.	Si consiglia di adoperare l'intera gamma di PPE (e.g. guanti, camici impermeabili a maniche lunghe).
TRASPORTO AL SITO DESTINATO ALLA CONSERVAZIONE	Messa in atto delle procedure locali standard. Assenza di condizioni necessarie per il trasporto della salma in condizioni speciali. Decontaminazione del mezzo impiegato per il trasporto minimizzando i contati con le spoglie. In caso il trasporto implicasse lo spostamento in un altro paese, sarebbe necessaria la consultazione delle norme specifiche in materia inerente l'ammissione di salme di pazienti deceduti per COVID-19 sospetta/confermata.	Guanti, guanti monouso in nitrile, mascherine chirurgiche, mascherine sanitarie a triplo strato, camici impermeabili a maniche lunghe.
LUOGO ADDETTO ALLA CONSERVAZIONE DELLA SALMA	L'ambiente esterno dovrebbe essere mantenuto pulito e igienizzato. La struttura dovrebbe presentare ventilazione ed illuminazione adeguate.<br>Le celle frigorifere dovrebbero mantenere una temperatura costante di 4°C.<br>Andrebbero messe in pratica le misure di smaltimento in sicurezza di eventuale materiale organico.	Il contatto diretto con le spoglie od eventuali fluidi corporei va minimizzato nell'atto del ricevimento della salma presso il sito addetto alla conservazione. Una rigida osservanza all'utilizzo dei PPE e delle misure precauzionali (e.g. guanti, camici impermeabili a maniche lunghe, igiene delle mani, occhiali protettivi/protezioni per la faccia, mascherine). Le procedure generanti aerosol non dovrebbero essere consentite.
ESPOSIZIONE DELLA SALMA	La salma va esposta in una zona pre-determinata accessibile alle persone care piu' vicine. Si raccomanda la messa in atto di precauzioni universali nel momento in cui la salma viene esposta. Alle persone care al defunto vanno fornite adeguate direttive al fine di assicurare un utilizzo corretto dei PPE. Si raccomanda di far desistere i parenti da ogni eventuale contatto diretto con la salma.	I PPE non sono necessari per la visita dei familiari, anche se questi ultimi devono evitare ogni eventuale contatto con la salma. Nel caso i parenti dovessero toccare le spoglie, si devono implementare le procedure raccomandate di igiene individuale.
IMBALSAMAZIONE	L'imbalsamazione della salma è, per lo più, non raccomandata, tuttavia, nel caso dovesse essere effettuata, si raccomanda di coinvolgere personale adeguatamente preparato a questa operazione e munito di validi PPE : le procedure di sicurezza standard dovranno essere seguite	Igiene delle mani ed uso dei PPE standard (e.g. guanti e camici impermeabili a maniche lunghe) se la procedura è consentita
SEPOLTURA	La sepoltura può essere effettuata regolarmente. Il personale addetto alla deposizione della bara va adeguatamente educato in merito alla COVID-19 ed alle relative misure precauzionali.	I requisiti minimi includono l'utilizzo di guanti e camici impermeabili a maniche lunghe per il personale addetto alla sepoltura . Va evitata la formazione di assembramenti durante le funzioni funebri e si consiglia di ricordare ai partecipanti di mantenere distanziamento sociale e di lavarsi le mani.
CREMAZIONE	La cremazione può essere effettuata in caso di disponibilità da parte delle apposite strutture. La salma va cremata direttamente entro le 24 ore dal decesso senza passaggio in ambito domestico. Il personale addetto alla procedura va adeguatamente educato. Alle persone care è concessa la visione delle ceneri. Si mantengono inalterate ulteriori procedure locali.	Si consiglia l'osservanza delle misure precauzionali e di protezione. Va evitata ogni forma di assembramento presso il crematorio.
PIANIFICAZIONE	Vanno stabiliti dei piani di emergenza circa il trattamento delle salme, l'accesso e la disponibilità di personale qualificato, disponibilita' di trasporti specializzati, di equipaggiamento specifico ed di siti di conservazione delle spoglie. Si consiglia la valutazione dei programmi esistenti e la discussione concordata della risposta da attuare.	Valutazione della necessità di PPE da parte dei lavoratori sanitari e l'eventuale bisogno di uno specifico addestramento circa il loro utilizzo.
ALTRO	Consultazione con le persone coinvolte in queste procedure (per esempio eventuali ministri di culto) per validare l'accettabilità dei cambiamenti alle pratiche funebri abituali. Va fornito supporto psicologico ai familiari dei defunti.	

Abbreviazioni: PPE=Dispositivi da protezione individualeRADE=Grading of Recommendations, Assessment, and Evaluation.

Contatto: Dr Elie Akl | ea32@aub.edu.lb

1866 AUB American University of Beirut الجامعة الأميركية في بيروت McMaster University Michael G. DeGroote COCHRANE CANADA CENTRE GRADE Centre | McMaster University

cc BY NC

Manejo seguro de corpos de pacientes com suspeita ou confirmação de infecção por COVID-19

Revisão sistemática rápida para a Organização Mundial de Saúde

ETAPAS ABORDADAS	MANEJO DE CORPOS	EPI DE INDIVÍDUOS ENVOLVIDOS NO MANEJO DOS CORPOS
PREPARAÇÃO DO CORPO	Remover acessos intravenosos e linhas arteriais.<br>Desinfetar orifícios de punção.<br>Desinfetar as superfícies onde o corpo estava.<br>Manejar como categoria de risco específica.<br>Precauções gerais com os materiais utilizados.	Luvas, capote/avental impermeável com mangas compridas, máscaras, óculos, limitar o envolvimento apenas de profissionais diretamente envolvidos.
EMPACOTAMENTO	Cobrir o corpo com sacos/bolsas de camada dupla e a prova de vazamento.<br>Desinfetar a embalagem externa.	Complemento completo dos EPI (por exemplo, luvas e capote/avental impermeável com mangas compridas).
TRANSPORTE PARA O LOCAL DE ARMAZENAMENTO	Procedimento de rotina local padrão. Nenhum transporte especial é necessário.<br>Descontaminar o veículo e minimizar o contato com restos humanos.<br>Se mudar de país, verificar restrições à importação de corpos com suspeita de infeção ou infecção confirmada por COVID-19.	Luvas, luvas descartáveis de nitrilo, máscara cirúrgica, máscara médica de camada tripla,<br>capote/avental impermeável com mangas compridas.
LOCAL DE ARMAZENAMENTO	Deve permanecer limpo e desinfetado.<br>Instalações adequadamente ventiladas e iluminadas.<br>Câmaras frias a 4°C.<br>Praticar o descarte seguro de resíduos.	O contato direto com restos humanos ou fluidos corporais deve ser minimizado durante a recepção em locais específicos para o armazenamento do corpo. Procedimentos rigorosos de EPI (por exemplo, luvas, capote/avental, capote/avental impermeável com mangas compridas, higiene das mãos óculos / protetor facial, máscaras). Nenhum procedimento com geração de aerossóis deve ser permitido.
VELÓRIO	Somente por parentes próximos em áreas pré-designadas.<br>Utilizar as precauções padrões para permitir a visualização do corpo.<br>Fornecer suporte sobre uso apropriado de EPI para os entes em luto. Aconselha-los a não tocar no corpo.	O uso EPI por parentes não é necessário; no entanto, eles devem ter qualquer contato com o corpo limitado. Se os parentes tiverem tocado o corpo, utilizar os procedimentos de higiene preconizados.
EMBALSAMENTO	Majoritariamente não recomendado. Se realizado, utilizar precauções padrões e medidas de proteção, e somente por equipe treinada.	Higiene das mãos e uso de EPI padrões (por exemplo, luvas e capote/avental impermeável com mangas compridas), se o procedimento for permitido.
SEPULTAMENTO	Pode ser realizado regularmente. Sensibilizar a equipe do enterro sobre o COVID-19 e as precauções padrões.<br>Rituais religiosos são permitidos desde que não haja contato com o corpo.	Os requisitos mínimos incluem luvas e capote/avental impermeável com mangas compridas para a equipe.<br>Evitar aglomerações durante os serviços funerários e aconselhar os participantes a manter distanciamento físico e higiene das mãos.
CREMAÇÃO	Pode ser realizado se houver instalações disponíveis.<br>Cremar em até 24 horas, sem levar o corpo para casa.<br>Sensibilizar a equipe. Parentes podem ver as cinzas de cremação. Seguir os procedimentos locais.	Seguir as medidas de proteção e precauções padrões.<br>Evitar aglomerações no crematório.
PLANEJAMENTO	Estabelecer um plano de preparação para manejo dos corpos, acesso a equipe treinada, transporte, equipamento e armazenamento dos corpos. Revisar os planos nacionais de contingência e comunicá-los.	Avaliar a necessidade do uso de EPI por profissionais de saúde e a necessidade de treinamento sobre seu uso.
OUTROS	Consultar as partes interessadas (por exemplo, representantes religiosos) para garantir a aceitabilidade das mudanças na prática padrão.<br>Fornecer aconselhamento aos membros da família.	

Abreviações: EPI=Equipamento de proteção individual; GRADE=Grading of Recommendations, Assessment, and Evaluation.
Contato: Dr Elie Akl | ea32@aub.edu.lb

1866 AUB American University of Beirut الجامعة الأميركية في بيروت
McMaster University
Michael G. DeGroote COCHRANE CANADA CENTRE
GRADE Centre | McMaster University

cc BY NC

Appendix 4: Expanded version of the infographic summarizing the steps reported in the guidance documents.

Safe Management of Bodies of Deceased Persons with Suspected or Confirmed COVID-19

Rapid Systematic Review

The 2019 novel coronavirus (COVID-19) pandemic has led to an exponentially growing number of deaths. There is an urgent need for proper strategies to minimize the risk of infection in individuals handling the bodies of deceased persons infected with COVID-19.

We performed a systematic review of the literature to first scope, and then assess the effects of specific strategies for the management of the bodies of deceased persons with suspected or confirmed COVID-19 infection.

We performed a search (of randomized and non-randomized studies without language restriction) for:

A) Guidance documents providing practical advice on the handling of bodies of deceased persons with suspected or confirmed COVID-19 infection.

B) Direct evidence on COVID-19.

C) Indirect evidence on the severe acute respiratory syndrome (SARS) virus and the Middle East Respiratory Syndrome (MERS) virus.

Results

We identified 23 guidance documents providing practical advice on the steps for handling the bodies of deceased persons with suspected or confirmed cases of COVID-19.

We did not identify COVID-19 evidence relevant to any of these steps. We identified one study that proposed an approach for handling autopsies of patients with SARS. The study provided very low certainty evidence that the proposed approach reduced the risk of transmission to the personnel handing the dead bodies.

STEPS ADDRESSED AND MAIN POINTS OF GUIDANCE		PPE
BODY PREPARATION	Remove IV tubes and lines.<br>Disinfect puncture holes.<br>Disinfect surfaces where body was resting<br>Manage as a specific risk category.<br>Universal precautions of material used.	Gloves, long-sleeved water resistant gown, surgical mask or N95, goggles, limit direct personnel involvement.
PACKING	2-layer cover of the body, leak-proof, bag, with a double layer cloth sheet soaked in disinfectant.<br>First layer is transparent, 2nd layer opaque.<br>Disinfect outer packing.	Gloves, long-sleeved water resistant gown, goggles, face-shield, mask, gloves, cover-all/gowns (with or without aprons), head cover and shoe cover should be worn (classified as high risk procedure).
TRANSPORT TO STORAGE SITE	Standard local routine procedure. No special transport required. Decontaminate vehicle after use. Minimize direct contact with human remains.	Gloves, disposable nitrile gloves, surgical mask, triple layer medical mask, long-sleeved water-resistant gown, PPE not otherwise specified.<br>Transport to mortuary classified as low risk procedure.
STORAGE SITE	Should remain clean daily and disinfected. Properly ventilated and illuminated. Cold chambers at 4°C. Practice safe waste disposal.	Direct contact with human remains or bodily fluids should be minimized during reception at designated body storage sites. Strict PPE procedures, gloves, gown, long-sleeved water-resistant gown, hand hygiene, goggles/face shield, medical or surgical or N95 mask. No aerosol generating procedures should be allowed.
VIEWING	Only by close relatives in a pre-designated area. Use standard precautions to allow the view of the body. Provide support for mourners in appropriate PPE use. Advise mourners not to touch the body.	PPE is not needed for relative viewing, yet, they should limit any contact with the body. If relatives have touched the body, use recommended hygiene procedures.
EMBALMING	Not recommended. If performed, use standard precautions and protective measures, and only by trained personnel.	Standard PPE as above if the procedure is allowed.
BURIAL	Can be performed regularly. Sensitize burial staff about COVID-19 and standard precautions. Religious rituals allowed as long as there is no contact with the body.	The minimum requirements include gloves and long-sleeved water-resistant gown for staff.<br>Avoid any crowding during funeral services, and advice participants to maintain physical distancing.
CREMATION	Advisable. Can be performed regularly if facilities are available. Cremate within 24 hours without taking the body home. Sensitize staff. Relatives can view dust. Follow local procedures.	PPE is not necessary for managing dust.
ADMINISTRATIVE MEASURES	Establish preparedness plan for handling dead bodies, access to trained staff, transport, equipment, and body storage. Review national contingency plans and communicate.	Assess and manage the need for PPE in healthcare professionals as well as the increased number of dead bodies.
OTHER	Consult with stakeholders (e.g. religious representatives) to ensure acceptability of changes to standard practice.<br>Provide counselling to family members.	

Conclusions

We present guidance documents that propose specific strategies for the management of the bodies of deceased persons with suspected or confirmed COVID- 19 infection. We found only one small study that describe one strategy for SARS infection but none for COVID-19. While this review highlights the need for research in this field, it allows interested entities to build their own guidance based on the identified guidance documents.

Abbreviations: PPE=Personal Protective Equipment; GRADE=Grading of Recommendations, Assessment, and Evaluation.
Contact: Dr. Elie Akl | ea32@aub.edu.lb

References

1. Centers for Disease Control and Prevention. Collection and Submission of Postmortem Specimens from Deceased Persons with Known or Suspected COVID-19 (Interim Guidance) US: CDC; 2020 [updated March 25, 2020. Available from: https://www.cdc.gov/coronavirus/2019-ncov/hcp/guidance-postmortem-specimens.html].
2. Government of India Ministry of Health & Family Welfare Directorate General of Health Services. COVID-19: Guidelines on Dead Body Management India: 2020 [Available from: https://www.mohfw.gov.in/1584423700568_COVID19GuidelinesonDeadbodymanagement.pdf]
3. European Centre for Disease Prevention And Control. Considerations related to the safe handling of bodies of deceased persons with suspected or confirmed COVID-19 Stockholm ECDC; 2020 [Available from: https://www.ecdc.europa.eu/sites/default/files/documents/COVID-19-safe-handling-of-bodies-or-persons-dying-from-COVID19.pdf]
4. Centre for Health Protection. Precautions for Handling and Disposal of Dead Bodies Hong Kong: CHP; 2020 [Available from: https://www.chp.gov.hk/files/pdf/grp-guideline-hp-ic-precautions_for_handling_and_disposal_of_dead_bodies_en.pdf]
5. Solna Publich Health Agency. Rekommendationer för hantering av avliden med bekräftad COVID-19 Sweden2020 [Available from: https://www.folkhalsomyndigheten.se/contentassets/4a38594abfec482c9b2b7fbedab7f9e3/omhandertagande.pdf].
6. World Health Organization. Infection Prevention and Control for the safe management of a dead body in the context of COVID-19: WHO; 2020 [Available from: https://apps.who.int/iris/handle/10665/331538].
7. Zhejiang University School of Medicine. Handbook of COVID-19 Prevention and Treatment China FAZHU; 2020 [Available from: http://www.zju.edu.cn/english/2020/0323/c19573a1987520/page.htm].
8. Ministry of Health Srilanka. Provisional Clinical Practice Guidelines on COVID-19 suspected and confirmed patients Srilanka: MoH 2020 [Available from: https://www.president.gov.lk/provisional-clinical-practice-guidelines-on-covid-19-suspected-and-confirmed-patients/].
9. European Centre for Disease Prevention and Control. Infection prevention and control for COVID-19 in healthcare settings United Kingdom: ECDC; 2020 [Available from: https://www.ecdc.europa.eu/en/publications-data/infection-prevention-and-control-covid-19-healthcare-settings].
10. Public Health England. Guidance for first responders and others in close contact with symptomatic people with potential COVID-19 United Kingdom2020 [Available from: https://www.gov.uk/government/publications/novel-coronavirus-2019-ncov-interim-guidance-for-first-responders/interim-guidance-for-first-responders-and-others-in-close-contact-with-symptomatic-people-with-potential-2019-ncov#handling-the-deceased].
11. Estonian Health Board Ministry of Social Affair. Käitumisjuhend COVID-19 nakkusega või nakkuskahtlusega surnu korral Estonia2020 [Available from: https://www.terviseamet.ee/sites/default/files/Nakkushaigused/Juhendid/COVID-19/surnukeha_kasitlemise_pohimotted_haiglates.pdf].
12. Ministry of Health and Family Welfare. Novel Coronavirus Disease 2019 (COVID-19): Guidelines on rational use of Personal Protective Equipment Bangladesh 2020 [Available from: https://www.mohfw.gov.in/pdf/GuidelinesonrationaluseofPersonalProtectiveEquipment.pdf].

13. Health Protection Scotland. COVID-19 guidance for infection prevention and control in healthcare settings Scotland2020 [Available from: https://www.hps.scot.nhs.uk/web-resources-container/covid-19-guidance-for-infection-prevention-and-control-in-healthcare-settings/].
14. Centre for Respiratory Diseases and Meningitis and Outbreak Response. Coronavirus disease 2019 (COVID-19) caused by a Novel Coronavirus (SARS-CoV-2): Guidelines for case-finding, diagnosis, management and public health response in South Africa. South Africa: 2020 [Available from: https://www.nicd.ac.zawp-content/uploads/2020/03/NICD_DoH-COVID-19-Guidelines-10March2020_final.pdf].
15. Partners in Health. COVID-19 Part II: Clinical Management of COVID-19: PIH; 2020 [updated March 21 2020. 49]. Available from: https://www.pih.org/sites/default/files/2020-03/PIH_Guide_COVID_Part_II_Clinical_Management_3_21.pdf].
16. New South Wales Government. Infection Prevention and Control COVID-19 (SARS-CoV-2) – Residential & Aged Care Facilities 2020 [updated March 12 2020. 21]. Available from: http://cec.health.nsw.gov.au/__data/assets/pdf_file/0007/571543/RACF-Infection-prevention-and-control-COVID-19-Interim-guidance-March-2020.pdf.
17. New South Wales Government. Infection Prevention and Control COVID-19 (SARS-CoV-2) – Hospital setting 2020 [updated March 12 2020. 21]. Available from: https://aci.health.nsw.gov.au/__data/assets/pdf_file/0003/568506/Infection-control-nCoV-2019-Hospital-Setting-V2-.pdf].
18. The Royal College of Pathologists. Autopsy practice relating to possible cases of COVID-19 (2019-nCov, novel coronavirus from China 2019/2020) (Briefing). 2020 [updated 24 March 2020. Available from: https://www.rcpath.org/uploads/assets/d5e28baf-5789-4b0f-acecfe370eee6223/fe8fa85a-f004-4a0c-81ee4b2b9cd12cbf/Briefing-on-COVID-19-autopsy-Feb-2020.pdf].
19. Anup Agarwal NN, Pranab Chatterjee, Swarup Sarkar, Devendra Mourya, Rima Rakeshkumar Sahay & Rajesh Bhatia. Guidance for building a dedicated health facility to contain the spread of the 2019 novel coronavirus outbreak. Indian Journal of Medical Research. 2020.
20. Chinese Medical Association Association of Pathologists. [Provisional guidelines on autopsy practice for deaths associated with COVID-19]. Chinese Journal of Pathology. 2020.
21. Mao Dan-mi ZN, Zheng Da, LE Jia-cheng, Zhao Qian-hao, Luo Bin, Guan Da-wei, Zhou Yi-wu, Hu Bing-jie, Cheng Jian-ding. [Guide to the Forensic Pathology Practice on Death Cases Related to Corona Virus Disease 2019(COVID-19)]. Jounal of Forensic Medicine 2020;36(1).
22. Sundhedsstyrelsen. Retningslinjer for håndtering af COVID-19 i sundhedsvæsenet: Guidelines for handling COVID-19 in health care Denmark: 2020 [Available from: https://www.sst.dk/-/media/Udgivelser/2020/Corona/Retningslinjer-marts/Retningslinje-COVID-19.ashx?la=da&hash=84942068EA39162A0B8E26A6AAFD6CC256276F14].
23. Helsedirektoratet. Koronavirus – beslutninger og anbefalinger. Norway: 2020 [Available from: https://www.helsedirektoratet.no/veiledere/koronavirus].
